# A dual-time-window protocol to reduce acquisition time of dynamic tau PET imaging using [^18^F]MK-6240

**DOI:** 10.1186/s13550-021-00790-x

**Published:** 2021-05-27

**Authors:** Guilherme D. Kolinger, David Vállez García, Talakad G. Lohith, Eric D. Hostetler, Cyrille Sur, Arie Struyk, Ronald Boellaard, Michel Koole

**Affiliations:** 1 Medical Imaging Center, University Medical Center Groningen, University of Groningen, Hanzeplein 1, 9713 GZ Groningen, The Netherlands; 2 Translational Imaging Biomarkers, Merck & Co., Inc., 770 Sumneytown Pike, Mailstop WP44D-216, West Point, PA 19486 USA; 3 Translational Pharmacology, Merck & Co., Inc, 351 N Sumneytown Pike, Mailstop UG4D-48, North Wales, PA 19454 USA; 4 Department of Radiology and Nuclear Medicine, Amsterdam University Medical Center, Location VU Medical Center, De Boelelaan 1117, 1081 HV Amsterdam, The Netherlands; 5 Nuclear Medicine and Molecular Imaging, Department of Imaging and Pathology, KU Leuven, Herestraat 49 – Bus 7003, 3000 Leuven, Belgium

**Keywords:** [18F]MK-6240, Dual-time-window, PET quantification, Reference logan, Tau imaging

## Abstract

**Background:**

[^18^F]MK-6240 is a PET tracer with sub-nanomolar affinity for neurofibrillary tangles. Therefore, tau quantification is possible with [^18^F]MK-6240 PET/CT scans, and it can be used for assessment of Alzheimer’s disease. However, long acquisition scans are required to provide fully quantitative estimates of pharmacokinetic parameters. Therefore, on the present study, dual-time-window (DTW) acquisitions was simulated to reduce PET/CT acquisition time, while taking into consideration perfusion changes and possible scanning protocol non-compliance. To that end, time activity curves (TACs) representing a 120-min acquisition (TAC_120_) were simulated using a two-tissue compartment model with metabolite corrected arterial input function from 90-min dynamic [^18^F]MK-6240 PET scans of three healthy control subjects and five subjects with mild cognitive impairment or Alzheimer’s disease. Therefore, TACs corresponding to different levels of specific binding were generated and then various perfusion changes were simulated. Next, DTW acquisitions were simulated consisting of an acquisition starting at tracer injection, a break and a second acquisition starting at 90 min post-injection. Finally, non-compliance with the PET/CT scanning protocol were simulated to assess its impact on quantification. All TACs were quantified using reference Logan’s distribution volume ratio (DVR) and standardized uptake value ratio (SUVR_90_) using the cerebellar cortex as reference region.

**Results:**

It was found that DVR from a DTW protocol with a 60-min break between two 30-min dynamic scans closely approximates the DVR from the uninterrupted TAC_120_, with a regional bias smaller than 2.5%. Moreover, SUVR_90_ estimates were more susceptible (regional bias ≤ 19%) to changes in perfusion compared to DVR from a DTW TAC (regional bias ≤ 10%). Similarly, SUVR_90_ was affected by late-time scanning protocol delays reaching an increase of 8% for a 20-min delay, while DVR was not affected (regional bias < 1.5%) by DTW protocol non-compliance.

**Conclusions:**

Therefore, such DTW protocol has the potential to increase patient comfort and throughput without compromising quantitative accuracy and is more reliable against SUVR in terms of perfusion changes and protocol deviations, which could prove beneficial for drug effect assessment and patient follow-up using longitudinal [^18^F]MK-6240 PET imaging.

**Supplementary Information:**

The online version contains supplementary material available at 10.1186/s13550-021-00790-x.

## Background

Accumulation of neurofibrillary tangles (NFT) is related to cognitive decline and is one of the neuropathological hallmarks of Alzheimer’s disease (AD) [1]. Furthermore, AD progression has been shown to follow a specific pattern described by the Braak Stages [2] such that *in-vivo* quantification of NFTs may support disease staging and assessing the neurological condition of patients. This can be achieved with positron emission tomography (PET) tracers that bind to hyper-phosphorylated tau proteins, such as [^18^F]MK-6240 which has sub-nanomolar affinity for NFTs and has been studied and validated recently in healthy control (HC), mild cognitive impairment (MCI), and AD subjects [3–7].

Previous in-human [^18^F]MK-6240 studies performed dynamic PET scans with a duration up to 180 min and used a reference tissue model with the cerebellum as reference brain region. This reference tissue model approach uses the time activity curve (TAC) of a reference tissue assumed to be devoid of specific tracer binding to estimate the ratio of specific to non-displaceable binding in target regions. This way, arterial cannulation for blood collection during the PET scan is obviated, avoiding an invasive and uncomfortable procedure, which often is not feasible in ill-conditioned patients. However, the long PET scanning times increases patient discomfort and the risk of head motion, which could hamper accurate quantification, and long scanning times are impractical for clinical routine scheduling. To address the issues of long dynamic PET scanning, one could consider standardized uptake value ratio (SUVR) relative to the cerebellum of a late static PET scan to estimate specific [^18^F]MK-6240 binding. A previous study [4] proposed static scanning at 90 min post-injection (p.i.), while another study [5] found that SUVR was still increasing at 135 min p.i. in subjects with high [^18^F]MK-6240 binding. In addition to the uncertainty about the optimal time-window for static [^18^F]MK-6240 scanning, SUVR can be susceptible to blood–brain barrier (BBB) functionality changes and variations in cerebral blood flow (CBF). It has been shown that such changes can introduce a bias to SUVR-based longitudinal assessment of deposition of β-amyloid plaques [8–10], another AD neuropathological hallmark. It can also be expected that drug intervention may have effects on perfusion. Given the high BBB permeability and fast plasma clearance of [^18^F]MK-6240 [6, 11, 12], one can also expect the influence of those factors and regional CBF functionalities to affect tau quantification. Thus, SUVR-based quantification should be considered carefully, notably in longitudinal settings for cases where the vascular component is expected to play an important role, such as for stroke patients or patients with cognitive decline. Furthermore, tau pathology and changes in relative CBF have been observed to independently contribute to cognitive deficits in AD [13], emphasizing the need to understand the interplay between tau load, tracer binding, and longitudinal changes in CBF. Nevertheless, SUVR from [^18^F]MK-6240 has potential for stratification of different stages of tau accumulation in a cross-sectional setting [7].

To avoid the limitations of SUVR quantification, two studies implemented a dual-time-window (DTW) protocol consisting of first dynamic PET scan starting at tracer injection, followed by a short resting period of 15 min [5] or 30 min [6], and a second dynamic PET scan starting after the short break. However, these studies did not evaluate the potential impact of a DTW protocol on the PET quantification nor did they optimize the scan and break duration of their DTW protocol. For amyloid quantification, there has been an evaluation of the effects of a DTW and CBF changes [14, 15]; however, no such exploration has been done for tau tracers.

The aim of the present study was to develop and evaluate a DTW protocol for accurate [^18^F]MK-6240 PET quantification that is comfortable for patients, suitable for longitudinal studies and drug intervention assessment, and appropriate for clinical routine. For this purpose, DTW protocols combining a first dynamic PET scan of variable length, starting at tracer injection, with a second 90- to 120-min PET scan were compared to an uninterrupted 120-min dynamic PET scan using a Logan reference tissue model (Ref Logan) with grey matter cerebellum as reference region. Furthermore, changes in perfusion were simulated to assess the impact of CBF and BBB permeability variations on Ref Logan and SUVR quantification, where SUVR was calculated relative to the grey matter cerebellum for the 90- to 120-min PET scan. Finally, small delays for the 90- to 120-min PET scan were simulated to evaluate the quantitative impact of possible non-compliance with the clinical routine protocol.

## Methods

### Subjects and image acquisition

PET and MR data of eight subjects were used for this study, three healthy controls (HC) and five patients classified with either Mild-Cognitive Impairment or Alzheimer’s Disease (MCI/AD). HC subjects had a Mini-Mental State Examination (MMSE) score of at least 27 and presented no cognitive complaints, MCI subjects had an MMSE score of at least 26 while presenting episodic memory impairment and a positive amyloid PET examination, and AD subjects had an MMSE score lower than 28 and were clinically diagnosed as probable AD based on the National Institute of Neurologic and Communicative Disorders and Stroke and of the Alzheimer Disease and Related Disorders Association criteria. Subject demographics are given in Table 1. The study was conducted at the University Hospital Leuven, Belgium, and approved by the local Ethics Committee (University Hospitals Leuven/KU Leuven, ClinicalTrials.gov no. NCT02562989). The subjects (or legal representative) gave written informed consent before enrollment in the study.

**Table 1:** Subject demographics

Subject	Clinical diagnosis	Sex	Age (y)	MMSE score	Dose (MBq)	Arterial sampling
S1	HC	F	59	29	161	Yes
S2	HC	M	66	29	153	Yes
S3	HC	M	68	29	155	Yes
S4	AD	M	67	11	160	Yes
S5	MCI	M	74	28	162	Yes
S6	MCI	M	80	27	158	Yes
S7	AD	M	74	13	163	No
S8	AD	F	70	13	157	No

Dynamic 90-min PET/computed tomography (CT) scans were acquired in 3D list mode on a Hirez Biograph 16 PET/CT camera (Siemens Medical Solutions) after intravenous bolus injection of [^18^F]MK-6240 (159 ± 3.3 MBq). A low-dose CT was acquired at the beginning of the PET/CT scan for attenuation correction. The dynamic acquisitions consisted of 31 frames (6 × 10, 6 × 20, 2 × 30, 2 × 60, 2 × 120, 10 × 300 and 3 × 600 s) and were reconstructed with a 3D filtered backprojection algorithm including correction for scatter, attenuation, decay, random coincidences, and dead time. A Gaussian post-reconstruction filter of 5 mm was applied, and the images had a final voxel size of 2.14 × 2.14 × 2.0 mm^3^ with a matrix size of 128 × 128 × 82. All subjects underwent a structural high-resolution T1-weighted MRI scan on a 3 T MR scanner (Philips Healthcare).

Manual arterial blood sampling was acquired for three HC and three MCI/AD subjects at 10 s intervals for the first 100 s of the PET/CT scan and approximately 2, 2.5, 3.5, 15, 30, 45, 60, 75, and 90 p.i.. These arterial blood samples were used to determine radioactivity levels in whole-blood and fractions of intact tracer in plasma as a function of time, such that a metabolite corrected arterial input function (AIF) was obtained for six subjects. Parent fractions for S2 had γ-counter measurement errors, therefore a HC-population-average parent fraction was used for this subject. Arterial cannulation failed two MCI/AD subjects (S7 and S8), and an AD-population-average arterial input function was used based on the average AIF of the three MCI/AD subjects with a measured AIF. Further details on the subject recruitment, radiotracer synthesis, PET/CT and MRI acquisitions, measurement of radiotracer in plasma, and parent fractions can be found elsewhere [6].

### PET image processing

Using PMOD (version 4.0, PMOD Technologies LLC), motion correction was performed on the 90-min dynamic PET data by aligning all frames with the average PET signal of the first five minutes of the dynamic PET scan using a normalized mutual information algorithm to determine the optimal rigid transformation. Next, PET data were co-registered with the corresponding T1w MRI data by a rigid transformation, obtained by maximizing the normalized mutual information between the average PET signal of all frames with the MRI data. Tissue class probability maps for grey matter (GM), white matter (WM), and cerebrospinal fluid (CSF) were estimated from each subject’s T1w MRI using the Computational Anatomy Toolbox (CAT) 12 (Jena University Hospital, Department of Psychiatry and Neurology[16]), a toolbox extension for Statistical Parametric Mapping (SPM) version 12 (Wellcome Centre for Human Neuroimaging). Next, each subject’s T1w MRI was spatially normalized to Montreal Neurological Institute (MNI) space using the Unified Segmentation Method, a non-linear registration approach regularized by the subject’s tissue probability maps [17, 18].

Anatomical brain regions of interest were initially defined based on the Hammers’ atlas [19]. Next, these predefined brain regions were combined to create composite anatomical volumes of interest (VOI) that are related to the Braak stages of disease progression:

- VOI 1: Entorhinal cortex (related to Braak stage 1)
- VOI 2: Hippocampus (related to Braak stage 2)
- VOI 3: Fusiform Gyrus + Amygdala (related to early Braak stage 3)
- VOI 3+: Medial part of the Anterior Temporal Lobe (related to advanced Braak stage 3)
- VOI 4: Lateral part of the Anterior Temporal Lobe + Medial and inferior temporal gyrus (related to Braak stage 4)
- VOI 5: All other brain regions except precentral and postcentral gyrus (related to Braak stage 5)
- VOI 6: Precentral and postcentral gyrus (related to Braak stage 6)

The cerebellum VOI from the Hammers’ atlas was masked with the grey matter tissue map (at 30% probability) to create the reference region.

Data analysis was carried in the MRI space of each subject, therefore the PET images were co-registered with their respective MRI. Furthermore, the predefined VOIs in MNI space were spatially transformed to the MRI subject space by the inverse nonlinear transformation used for the spatial normalization. The VOIs in MRI subject space were limited to grey matter by applying a threshold of 30% to the GM tissue probability map and were then projected on the 90-min dynamic PET data to extract the regional TACs.

### Dynamic PET simulations and quantification

#### Kinetic parameters

A three exponential function was fitted to the whole blood and plasma activity curves, while a Hill function was fitted to metabolite data. Next, a reversible two-tissue compartmental model (2TCM) was fitted to the regional TACs using the blood activity concentrations and metabolite corrected activity concentrations in arterial plasma as input function [6]. The blood volume was fixed to 5%, data points were weighted based on frame duration and the decay at frame mid-time relative to tracer injection, and the tissue TACs were corrected for blood delay. Based on the 2TCM kinetic parameters, TACs were extrapolated to 120 min length (TAC_120_). Distribution volume ratio (DVR) was obtained as the target-to-reference ratio of the total distribution volume (*V*_T_) from each tissue. Following, changes in perfusion were simulated by altering the *K*_1_ (tracer influx rate from plasma to tissue) and *k*_2_ (tracer clearance rate from tissue to plasma) parameters from the 2TCM. As such, *K*_1_ values were proportionally changed by ± 50%, ± 25%, and ± 10%, while *k*_2_ was varied accordingly to preserve the *K*_1_/*k*_2_ ratio constant. Whole-brain *K*_1_ changes were simulated by keeping *R*_1_ (= *K*_1_/*K*_1Cerebellum_) constant while region-specific *K*_1_ changes were simulated by altering only the *K*_1_ values for regions other than the cerebellum (thus with variable *R*_1_).

#### Dual-time-window protocol

Dual-time-window (DTW) acquisitions consist of two dynamic PET acquisitions separated by a break period in which the subjects may be allowed to move out of the PET scanner. Part 1 of the protocol is a dynamic PET scan starting at the time of the bolus injection of [^18^F]MK-6240. After the break, Part 2 is started, consisting of a 30-min dynamic PET scan starting 90 min p.i., which is in line with the current clinical protocol of a late time static scan. DTW acquisitions were simulated by removing the appropriate data points from TAC_120_ and TACs with changed *K*_1_ and *k*_2_, and then applying a linear interpolation to create the corresponding DTW TACs (TAC_DTW_). As such, four DTW protocols were evaluated:

1. 20-min PET scan + 70-min break + 30-min PET scan: TAC_DTW70_
2. 30-min PET scan + 60-min break + 30-min PET scan: TAC_DTW60_
3. 40-min PET scan + 50-min break + 30-min PET scan: TAC_DTW50_
4. 50-min PET scan + 40-min break + 30-min PET scan: TAC_DTW40_

In order to evaluate the impact of non-compliance with the DTW protocol, three additional delays for the start of Part 2 of the DTW protocol were simulated: 5 min, 10 min, and 20 min, while its duration remained fixed at 30 min. Therefore, the total length of the DTW scanning protocol was increased by the delay duration.

#### Quantitative and statistical analysis

All simulated TACs were analyzed with the Ref Logan model using the grey matter cerebellum as the reference region, assuming independence of the reference tissue clearance rate which requires a late equilibrium time, and therefore t* was fixed at 90 min. Furthermore, SUVR was calculated for each TAC using the 90–120 min interval p.i. (SUVR_90_), corresponding to Part 2 of the DTW protocol, as the average uptake in the target region relative to the average uptake in the cerebellar cortex. Impact of a delayed late time scan was evaluated for both Ref Logan and SUVR.

Inferential statistical analysis was performed to explore how changes to TAC_120_ (either changing perfusion, introducing a break, perfusion changes in a DTW acquisition, and considering scanning protocol non-compliance), affected Ref Logan DVR and SUVR. For that matter, the generalized estimating equations (GEE) model was used as it is known to achieve higher statistical power with small sample sizes than ANOVA and does not require data normality [20–22]. On the present study, the GEE model was built with a linear-scale response and an independent working correlation matrix. To compare DVR and SUVR (the dependent variables) of TACs with changed perfusion against themselves of TAC_120_ (with fitted *K*_1_ and *k*_2_), models using the full interaction of diagnosis, region, and *K*_1_ change as predictor factor (independent variables) were applied to the data. Meanwhile, for the assessment of the impact of the DTW break length in DVR estimation, a model using the full interaction of diagnosis, region, and break was fitted, and, finally, to study the impact of protocol non-compliance on DVR and SUVR, models with the full interaction between diagnosis, region, and delay were fitted to the data. All models were applied independently to the dependent variables: Ref Logan DVR and SUVR. The results of these models were reported with the relative percentage change alongside its 95% confidence interval, and a p value of 0.05 was used as the threshold for considering statistical significance (Wald method, without correction for multiple pairwise comparisons). Complete results of the GEE models were reported in the supplementary material. GEE analysis was carried out with R (version 3.6.3, Rstudio version 1.2.5033) using the packages geepack (version 1.3-1) and emmeans (version 1.4.7).

## Results

### Simulations of 120-min TAC

The average and standard deviation for the 2TCM parameters of TAC_120_ are given in Table 2.

**Table 2:** Average kinetic parameters from the two-tissue compartment model

Diagnosis	Region	Mean K_1_ (SD)	Mean k_2_ (SD)	Mean k_3_ (SD)	Mean k_4_ (SD)	Mean DVR (SD)	Mean V_T_ (SD)
HC	Cerebellum	0.402 (0.07)	0.151 (0.01)	0.0107 (0.002)	0.0160 (0.005)	-	4.63 (1.5)
	VOI 1	0.273 (0.04)	0.136 (0.01)	0.0173 (0.003)	0.0200 (0.007)	0.85 (0.72)	3.85 (0.9)
	VOI 2	0.301 (0.06)	0.124 (0.01)	0.0148 (0.003)	0.0264 (0.006)	0.84 (0.11)	3.80 (0.7)
	VOI 3	0.283 (0.04)	0.125 (0.004)	0.0138 (0.002)	0.0189 (0.006)	0.88 (0.10)	4.01 (0.9)
	VOI 3+	0.255 (0.04)	0.124 (0.003)	0.0155 (0.0003)	0.0157 (0.002)	0.91 (0.12)	4.11 (0.7)
	VOI 4	0.325 (0.05)	0.140 (0.01)	0.0139 (0.002)	0.0172 (0.005)	0.94 (0.07)	4.30 (1.1)
	VOI 5	0.362 (0.05)	0.160 (0.01)	0.0155 (0.002)	0.0198 (0.004)	0.90 (0.08)	4.10 (1.0)
	VOI 6	0.345 (0.05)	0.165 (0.01)	0.0168 (0.001)	0.0208 (0.004)	0.84 (0.09)	3.82 (0.9)
MCI/AD	Cerebellum	0.408 (0.09)	0.140 (0.01)	0.0198 (0.002)	0.0264 (0.007)	-	5.20 (1.0)
	VOI 1	0.243 (0.03)	0.102 (0.02)	0.0380 (0.006)	0.0147 (0.004)	1.78 (0.62)	9.31 (4.0)
	VOI 2	0.256 (0.04)	0.105 (0.02)	0.0343 (0.007)	0.0203 (0.008)	1.44 (0.57)	7.52 (3.6)
	VOI 3	0.240 (0.05)	0.089 (0.02)	0.0363 (0.010)	0.0142 (0.004)	2.02 (0.87)	10.54 (5.3)
	VOI 3+	0.223 (0.05)	0.089 (0.02)	0.0349 (0.007)	0.0157 (0.004)	1.71 (0.64)	8.79 (3.6)
	VOI 4	0.292 (0.09)	0.104 (0.03)	0.0353 (0.014)	0.0166 (0.005)	2.08 (1.26)	10.83 (7.0)
	VOI 5	0.329 (0.07)	0.140 (0.02)	0.0326 (0.009)	0.0208 (0.008)	1.36 (0.63)	7.09 (3.6)
	VOI 6	0.338 (0.06)	0.162 (0.02)	0.0321 (0.008)	0.0230 (0.010)	1.10 (0.51)	5.67 (2.6)

Two-tissue compartment model kinetic parameters for TAC_120_. Blood volume fraction was fixed at 5%. *K*_1_ represents the tracer influx from plasma to tissue, *k*_2_ the washout constant (tracer efflux from tissue to plasma), *k*_3_ is the transfer from the non-displaceable to specific compartment, *k*_4_ is the transfer from specific to non-displaceable compartment, DVR is the distribution volume ratio, and *V*_T_ is the total distribution volume. Alternatively, *k*_3_ and *k*_4_ can represent interactions between the free and the non-specific binding compartments for HC subjects. This interpretation is mathematically indistinguishable from the previously described *k*_3_ and *k*_4_. Average and standard deviation (SD) are shown for each parameter.

### Reference Logan DVR

DVR from Ref Logan calculated from TAC_120_ had a very strong correlation (*r*^2^ = 0.99) with the DVR estimated from 2TCM. Ref Logan DVR more closely approximated 2TCM DVR for NFT-poor regions than for NFT-rich target tissues, with the reference tissue model slightly underestimating 2TCM (DVR_Ref Logan_ = 0.75 × DVR_2TCM_ + 0.26; Additional file 1: Fig. S1).

#### Tracer perfusion changes

**Fig. 1:**
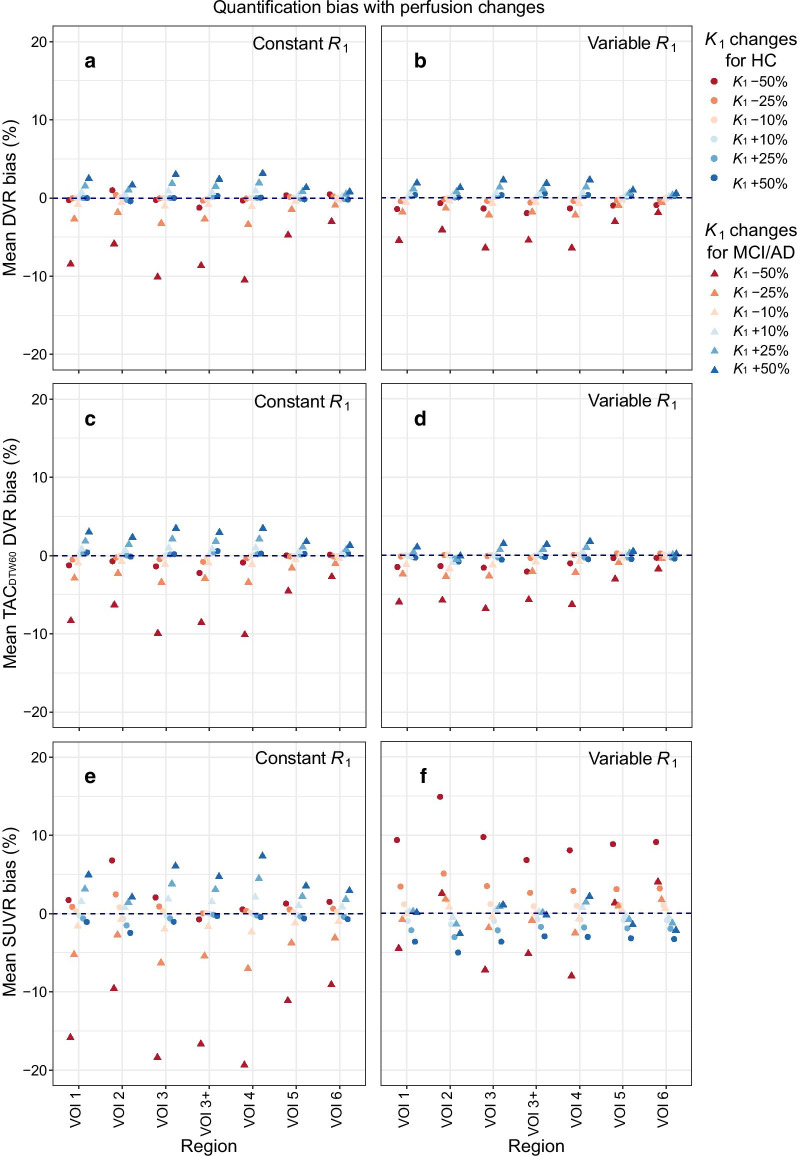
Average quantification bias due to perfusion changes. Changes with constant *R*_1_ are shown on the left, and with variable *R*_1_ on the right. Subject diagnosis is shown by data point shape and tracer influx rate (*K*_1_) changes by color. **a**, **b** show the bias for Reference Logan DVR on the uninterrupted TAC (TAC_120_), **c**, **d** show the bias for Reference Logan DVR on the dual-time-window TAC with 60-min break (TAC_DTW60_), and **e**, **f** show the bias for SUVR_90_

The DVR from TACs with perfusion changes and constant *R*_1_ showed, for HC subjects, an overall average bias (i.e., pooling target VOIs together) ranging from − 0.04% [− 0.35%:0.27%] (*p* = 0.789) with *K*_1_-50% to − 0.07% [− 0.16%:0.02%] (*p* = 0.137) with *K*_1_ + 50% when compared against the Ref Logan DVR of TAC_120_ with the fitted *K*_1_. Meanwhile, for MCI/AD subjects this bias ranged from − 4.7% [− 17.1%:1.5%] (*p* = 0.007) with *K*_1_-50% to 1.6% [− 0.9%:5.3%] (*p* = 0.019) with *K*_1_ + 50% (Additional file 2: Table S1). The largest regional bias for perfusion changes with constant *R*_1_ for HC subjects was − 1.2% [− 1.9%:− 0.5%] (*p* < 0.001) on VOI 3+, while for MCI/AD subjects it was − 10.5% [− 20.3%:− 0.7%] (*p* = 0.035) on VOI 4, in either case with *K*_1_-50% (Fig. 1a and Additional file 2: Table S2).

For perfusion changes with variable *R*_1_, the overall DVR bias for HC subjects ranged from − 1.2% [− 1.5%:− 1.0%] (*p* < 0.001) with *K*_1_-50% to 0.31% [0.22%:0.41%] (*p* < 0.001) with *K*_1_ + 50%, while for MCI/AD subjects it ranged from − 4.9% [− 8.5%:− 1.4%] (*p* = 0.007) with *K*_1_-50% to 1.7% [0.32%:3.1%] (*p* = 0.016) with *K*_1_ + 50% (Additional file 2: Table S3). Figure 1b and Additional file 2: Table S4 also show that the largest average regional DVR bias due to perfusion changes with relative *R*_1_ variability was − 2.0% [− 2.1%:− 1.8%] (*p* < 0.001, *K*_1_-50%, VOI 3+) for HC subjects, while for MCI/AD subjects it was − 6.4% [− 12.2%:− 0.6%] (*p* = 0.031, *K*_1_-50%, VOI 4). Notice that the average regional bias was less than 10% for any region as long as *K*_1_ was not changed more than 25% (either constant *R*_1_ or relative *R*_1_ changes), regardless of diagnosis.

#### DTW protocol

**Fig. 2:**
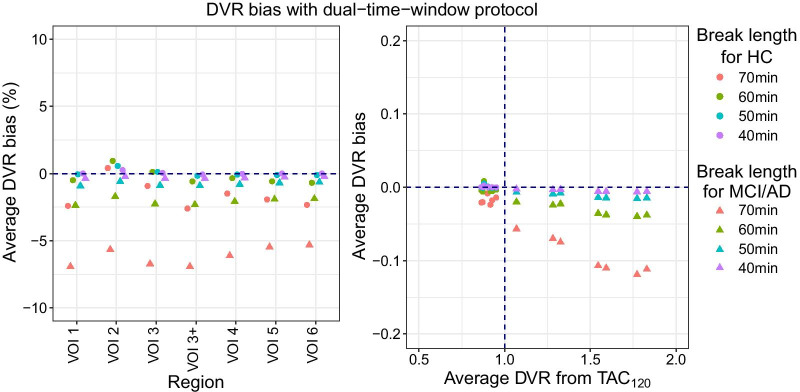
Average DVR bias from dual-time-window TACs when compared to the 120-min uninterrupted TAC (TAC_120_). The average relative bias for each region of interest is shown on the left panel, while the right panel shows the average DVR bias as function of DVR from TAC_120_. Diagnoses are indicated by data point shape and break length is indicated by color

Despite introducing breaks in the TACs, the DVR average regional differences between TAC_DTW_ and TAC_120_ were less than 10%, regardless of diagnosis and duration of the break (Fig. 2). When pooling all target VOIs, the overall average DVR bias for HC subjects was − 1.6% [− 2.6%:− 0.6%] (*p* = 0.002), − 0.2% [− 0.6%:0.2%] (*p* = 0.276), 0.03% [− 0.10%:0.16%] (*p* = 0.663) and 0.04% [− 0.0002%:0.08%] (*p* = 0.051) for a break length of 70 min, 60 min, 50 min and 40 min, respectively, while for MCI/AD subjects the overall average DVR bias was − 6.2% [− 8.97%:− 3.51%] (*p* < 0.001) for the 70-min break, − 2.1% [− 3.2%:− 1.0%] (*p* < 0.001) for the 60-min break, − 0.79% [− 1.2%:− 0.34%] (*p* = 0.001) for the 50-min break and − 0.31% [− 0.49%:− 0.13%] (*p* = 0.001) for the 40-min break (Additional file 2: Table S5). The longest break of 70 min (TAC_DTW70_) resulted in the largest DVR underestimation for both HC and MCI/AD subjects, with a regional average bias of − 2.6% [− 3.0%:− 0.2%] (*p* < 0.001, VOI 3+) and − 6.9% [− 9.6%:− 4.3%] (*p* < 0.001, VOI 3+), respectively (Additional file 2: Table S6). A slightly shorter break of 60 min (TAC_DTW60_) introduced a relatively low average bias (< 5%) for all brain regions when compared against TAC_120_, irrespective of diagnosis (Fig. 2 and Additional file 2: Table S6). Because of the low bias and the flexibility that a 60-min break provides, allowing for an interleaved scan of a secondary subject during the break of the primary subject, only this DTW protocol was considered for further analysis.

#### DTW protocol with tracer perfusion changes

Overall bias for perfusion changes on TAC_DTW60_ with constant *R*_1_ ranged from − 0.9% [− 1.5%:− 0.4%] (*p* = 0.001) with *K*_1_-50% to 0.3% [0.05%:0.45%] (*p* = 0.015) with *K*_1_ + 50% for HC subjects, while for MCI/AD subjects it ranged from − 7.7% [− 13.0%:− 2.3%] (*p* = 0.005) with *K*_1_-50% to 2.7% [0.83%:4.6%] (*p* = 0.005) with *K*_1_ + 50% (when compared against Ref Logan DVR from TAC_120_; Additional file 2: Table S7). The largest average regional DVR bias for HC subjects with TAC_DTW60_ and perfusion changes with constant *R*_1_ was − 2.2% [− 3.1%:− 1.01%] (*p* < 0.001, VOI 3+, *K*_1_-50%) and for MCI/AD subjects it was − 10.1% [− 19.3%:− 0.93%] (*p* = 0.031, VOI 4, *K*_1_-50%), as can be seen on Fig. 1c and Additional file 2: Table S8.

For perfusion changes with variable *R*_1_ on TAC_DTW60_, the overall DVR bias for HC subjects ranged from − 0.12% [− 1.4%:− 0.92%] (*p* < 0.001) with *K*_1_-50% to − 0.46% [− 0.57%:− 0.36%] (*p* < 0.001) with *K*_1_ + 50%, while for MCI/AD subjects it ranged from − 5.3% [− 9.4%:− 1.2%] (*p* = 0.012) with *K*_1_-50% to 0.99% [0.34%:1.7%] (*p* = 0.003) with *K*_1_ + 50% (when compared against Ref Logan DVR from TAC_120_; Additional file 2: Table S9). For HC subjects, the largest regional average DVR bias was − 2.1% [− 2.4%:− 1.8%] (*p* < 0.001, VOI 3+, *K*_1_-50%), while for MCI/AD subjects it was − 6.8% [− 12.4%:− 1.2%] (*p* = 0.017, VOI 3, *K*_1_-50%) (Fig. 1d and Additional file 2: Table S10).

#### Non-compliance with DTW protocol

**Fig. 3:**
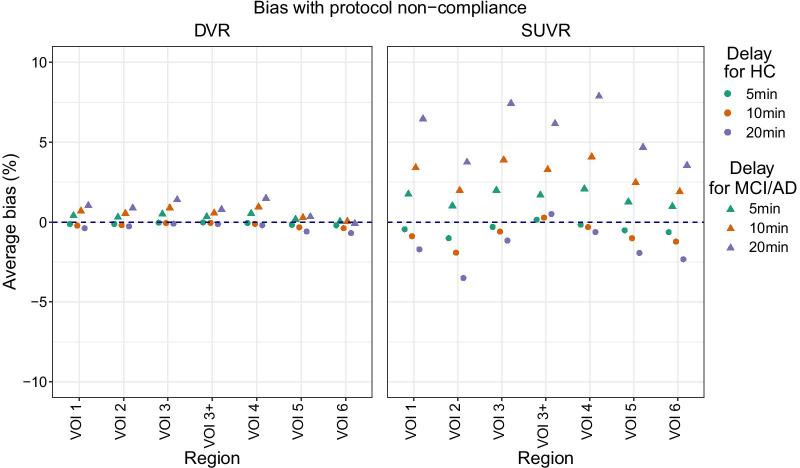
Ref Logan DVR bias (left) and SUVR_90_ bias (right) due to scan protocol non-compliance resulting in delays on the start of the late time point scan (i.e., Part 2 of the DTW protocol). Average bias was calculated for subjects with the same diagnosis, indicated by symbol shape. Delay length is indicated by colors

Delaying the start of Part 2 of the DTW protocol after a planned 60-min break, effectively increasing this break, only had a small impact on Ref Logan DVR estimates when compared to TAC_DTW60_ (Fig. 3). The overall bias for HC subjects was − 0.1% [− 0.19%:− 0.01%] (*p* = 0.036), − 0.19% [− 0.38%:− 0.002%] (*p* = 0.047), and − 0.33% [− 0.70%:0.03%] (*p* = 0.073) for 5, 10, and 20 min of delay, respectively. Meanwhile, for MCI/AD subjects this overall bias was 0.35% [− 0.12%:0.82%] (*p* = 0.140) for 5 min, 0.61% [− 0.28%:1.5%] (*p* = 0.178) for 10 min, and 0.92% [− 0.68%:2.5%] (*p* = 0.261) for 20 min of delay (Additional file 2: Table S11). The largest regional average DVR bias due to delays was − 0.69% [− 1.1%:0.29%] (*p* = 0.001) for HC subjects (VOI 6) and 1.5% [− 0.6%:3.5%] (*p* = 0.156) for MCI/AD subjects (VOI 4), both with a 20-min delay (Additional file 2: Table S12).

### SUVR quantification

Quantification with SUVR_90_ overestimated the DVR from both 2TCM and Ref Logan models (Additional file 3: Fig. S2). The level of overestimation was not constant throughout subjects and was not directly related to the regional SUVR_90_ value itself. Such behavior was independent of subject diagnosis; however, one AD patient had lower SUVR than 2TCM DVR in some of its target regions.

#### Tracer perfusion changes

Figure 1e, f shows the regional average bias for SUVR_90_ of TACs with changed perfusion relative to the SUVR_90_ of TAC_120_ (which has the fitted *K*_1_ and *k*_2_ values of each subject). SUVR_90_ of HC subjects was only slightly affected by perfusion changes with *R*_1_ constant, with overall bias ranging from 1.8% [1.3%:2.3%] (*p* < 0.001) for *K*_1_-50% to − 0.92% [− 1.1%:− 0.76%] (*p* < 0.001) for *K*_1_ + 50%. Meanwhile, for MCI/AD subjects the overall SUVR_90_ bias for perfusion changes with constant *R*_1_ ranged from − 15.1% [− 25.1%:− 5.1%] (*p* = 0.003) for *K*_1_-50% to 4.8% [0.78%:8.8%] for *K*_1_ + 50% (*p* = 0.019). The largest regional SUVR_90_ bias was 6.8% [5.2%:8.4%] (*p* < 0.001, VOI 2, *K*_1_-50%) and − 19.4% [− 37.7%:− 1.04%] (*p* = 0.038, VOI 4, *K*_1_-50%) for HC and MCI/AD subjects, respectively. More details can be found in the Additional file 2: Tables S13 and S14.

For perfusion changes with a variable *R*_1_, the overall SUVR_90_ bias ranged from 9.4% [5.9%:13.0%] (*p* < 0.001) with *K*_1_-50% to − 3.5% [− 4.7%:− 2.3%] (*p* < 0.001) with *K*_1_ + 50% for HC subjects, while for MCI/AD subjects this bias ranged from − 3.3% [− 11.0%:4.4%] (*p* = 0.399) with *K*_1_-50% to − 0.15% [− 3.0%:2.7%] (*p* = 0.916) with *K*_1_ + 50% (when compared against SUVR_90_ of TAC_120_). The largest regional SUVR_90_ bias for HC subjects was 14.9% [9.6%:20.2%] (*p* < 0.001, VOI 2, *K*_1_-50%), while for MCI/AD subject it was − 8.0% [− 21.9%:5.9%] (*p* = 0.260, VOI 4, *K*_1_-50%). More details can be found at the Additional file 2: Tables S15 and S16.

#### Non-compliance with late time static scan protocol

A delayed start of the late time static scans affected mainly the SUVR_90_ of MCI/AD subjects, in contrast to HC subjects (Fig. 3). Overall, SUVR_90_ of HC subjects was underestimated (when compared to the SUVR_90_ of the acquisition without delays) by − 0.39% [− 0.55%:− 0.23%] (*p* < 0.001), − 0.77% [− 1.1%:− 0.45%] (*p* < 0.001), and − 1.5% [− 2.1%:− 0.83%] (*p* < 0.001) for 5 min, 10 min, and 20 min delays, respectively. Meanwhile, the SUVR_90_ of MCI/AD subjects was, in overall, overestimated by 1.6% [0.38%:2.9%] (*p* = 0.010), 3.2% [0.70%:5.6%] (*p* = 0.012), and 6.0% [1.2%:10.9%] (*p* = 0.015) with 5-min, 10-min, and 20-min delays, respectively (Additional file 2: Table S17). More specifically, the largest regional SUVR_90_ bias for HC subjects was − 3.5% [− 4.1%:− 2.9%] (*p* < 0.001, VOI 2, delay of 20 min), while for MCI/AD subjects it was 7.9% [− 0.3%:16.1%] (*p* = 0.059, VOI 4, delay of 20 min). More details can be found at the Additional file 2: Tables S17 and S18.

## Discussion

For this study, a DTW PET protocol for [^18^F]MK-6240 PET imaging was considered while using a Ref Logan for DVR quantification, as well as the simulation of perfusion changes and scanning protocol non-compliance. As such, it was possible to reduce the overall acquisition time, therefore, potentially increasing patient comfort and clinical route feasibility while approximating a quantitative approach using full dynamic scanning. For the evaluation of different DTW protocols, dynamic PET data based on patient data was simulated up to 120-min post-injection and brain regions related to Braak Stages were considered. This way, realistic and clinically relevant datasets were generated for the regional assessment of a wide range of tau accumulation levels.

Presently, Ref Logan was assumed to be independent of the reference tissue clearance rate (*k*_2_’), which requires a late equilibrium time. To assess if the mismatch between 2TCM and Ref Logan for high binding subjects comes from the violation of this assumption, the 2TCM cerebellar *k*_2_ was used as *k*_2_’ on the Ref Logan model. This was done with a population-based *k*_2_’ as an average of the cerebellar *k*_2_ from all subjects, and with each individual’s cerebellar *k*_2_ as Ref Logan’s *k*_2_’. As there was no improvement on the agreement between the kinetic models (Additional file 1: Fig. S1), it is reasonable to trust the applicability of the Ref Logan model as independent of reference tissue clearance rate and indicates that the mismatch for high binding subjects is not a violation of this assumption.

Changes in perfusion had, in general, a significant impact on Ref Logan DVR estimations of MCI/AD subjects. It was observed that halving *K*_1_ lead to a regional DVR underestimation larger than 5% for most target regions, either when *R*_1_ was constant or variable (Fig. 1). Such extreme change in perfusion is not traditionally expected on longitudinal assessment of patients; however, these simulations were included to better understand general trends in quantification as function of perfusion changes and include possible extreme cases that could come from an interplay between disease progression and a possible drug effect. For less drastic perfusion changes, the average DVR bias was less than 3.5% for any target region, perfusion change (constant *R*_1_ or not), and subject diagnosis (Fig. 1). Therefore, such results indicate that a Ref Logan modelling using the cerebellar cortex as reference region can be a reasonable approach for longitudinal tau assessment of MCI and AD patients, or perhaps even for subjects undergoing treatment which may affect CBF and BBB functionalities. Furthermore, the simulation with variable *R*_1_ violates the assumption of reference tissue models that the ratio *K*_1_/*k*_2_ is the same for both target and reference tissues. As such, this simulation tested the robustness of the Ref Logan model and showed reliable quantification even when using a reference region that may have some PET signal (e.g. from spill-over, partial volume effects, or non-specific binding).

Regarding the introduction of a gap in the time-activity curves, it was demonstrated that a DTW protocol with a 60-min break between two 30-min dynamic acquisitions provided, on average, a bias of less than 2.5% when compared to a full dynamic scanning protocol (Fig. 2). This bias level is on the range of the expected test–retest variability [23–25] for AD studies and shorter breaks resulted in less quantification bias, which is in line with previous literature [14, 26, 27]. However, compared to shorter breaks, a 60-min break DTW protocol allows for efficient use of hospital facilities without compromising quantification accuracy since it still provides enough flexibility to schedule an interleaved clinical study while having low bias when compared against an uninterrupted 120-min scan. Additionally, by halving the total acquisition time, this DTW protocol (TAC_DTW60_) has the potential to decrease patient discomfort and, therefore, we considered this design as the DTW protocol of choice. Presently, a simple linear interpolation was performed to fill the gap in the TAC. Even though a previous study found that filling the DTW gap with more advanced techniques could improve quantification, linear interpolation still resulted in comparable results to those obtained from uninterrupted scanning [27]. Another scanning protocol option previously explored in the literature would be one uninterrupted dynamic PET scanning session of 60 min using the reference Logan plot to estimate the DVR values, as this approach provided low quantitative bias when compared to a more lengthy scanning session [5]. However, a scanning session of 60 min without a break could already be difficult to tolerate for patients suffering from significant cognitive decline. Moreover, the proposed DTW protocol is identical to other DTW protocols recommended for [^18^F]flutemetamol and [^18^F]florbetaben PET studies [14], which further facilitates its implementation as having the same protocol for different studies can simplify clinical workflow.

Next, the impact of changes in perfusion on the Ref Logan DVR quantification using the DTW 60-min break protocol was compared to the DVR results from the full dynamic scan analysis (TAC_120_). Furthermore, late time SUVR_90_ of TACs with perfusion changes were compared with the SUVR_90_ of the unaltered acquisition (fitted *K*_1_ and *k*_2_ parameters). It was observed that perfusion changes lead up to a regional DVR bias of 10% or less (Fig. 1), while for SUVR_90_ it was up to − 19%, showing that quantification of static images is more vulnerable to perfusion changes than the Ref Logan model. Ref Logan DVR from TAC_DTW60_ had a smaller range on the 95% confidence interval than SUVR90 (Additional file 2: Tables S8, S10, S14 and S16), showing that it can be more reliable against perfusion changes than SUVR. Furthermore, DVR dependence on perfusion with either constant or variable *R*_1_ was similar: lower perfusion (lower *K*_1_ values) decreased DVR of MCI/AD subjects and increased perfusion overestimated their DVR, while HC subjects were essentially unaffected (Fig. 1c, d). SUVR_90_ dependence on perfusion was different with constant *R*_1_ and variable *R*_1_. With constant *R*_1_, SUVR_90_ of MCI/AD subjects was directly related to perfusion (similar to what was observed on DVR estimates), while for HC subjects the SUVR_90_ had an opposite trend: increasing with lower perfusion and decreasing when perfusion increased (Fig. 1e). With variable *R*_1_, on the other hand, the SUVR_90_ variability for MCI/AD subjects was dependent of target region: on NFT-poor regions the behavior was similar to HC subjects, while on NFT-rich regions (high uptake VOIs: 1, 3, 3+, and 4) SUVR_90_ was essentially not affected (small effect size and *p* > 0.05) with exception of *K*_1_-50% changes, which showed larger differences when compared to SUVR_90_ from TAC with fitted *K*_1_. Furthermore, SUVR_90_ of HC subjects was increased with lower perfusion and decreased with higher perfusion, a similar trend when compared to perfusion changes with constant *R*_1_. Since reliable quantification requires a method that is unaffected by *K*_1_ and *k*_2_ changes, a DTW approach is preferred to SUVR_90_ quantification, since otherwise perfusion, plasma clearance, and BBB functionality changes can introduce a confounding factor for assessment of drug effects, follow-up studies, and disease staging, especially in diseases such as Alzheimer’s and stroke where vascular changes or changes in BBB permeability are to be expected.

Non-compliance with the scanning protocol led to an overestimation of the SUVR_90_ of MCI/AD subjects with longer delays, reaching an overall average overestimation of 6.0% [1.2%:10.9%] (*p* = 0.015) for 20 min of delay (Additional file 2: Table S17). This indicates that [^18^F]MK-6240 uptake is not in an equilibrium state after 140 min of uptake, which was also observed for high-binding subjects on a previous study [5] but contradicts the equilibrium found at 90 min p.i. by another group [4]. Without reaching an equilibrium, it is not possible to compare SUVR values estimated from different time windows and then deviations from the clinical protocol will lead to a bias on patient assessment. Since published studies have used different time windows for SUVR estimation, such as 60–90 min p.i. [6], 70–90 min p.i. [3, 5], 90–110 min [4, 7], and 90–120 min p.i. [5, 24], the SUVR values from these studies cannot be compared. However, SUVR at the 90–120-min window shows promising test–retest results for NFT-rich regions of AD subjects (6 ± 2%) and could possibly be a suitable metric for cross-sectional patient assessment [24] as long as there are no deviations from the scanning protocol. That study also found that NFT-poor regions presented higher test–retest variability (14 ± 6%). Contrary to SUVR, overall Ref Logan DVR was not impacted by scan delays (effect size < 1% regardless of diagnosis and delay length; Additional file 2: Table S11) and its use is recommended over SUVR for quantification on a time window in which tracer equilibrium has not been reached. It was observed, however, that the DVR of VOI 5 and VOI 6 were less impacted than other target regions of MCI/AD subjects (Fig. 3). That is a consequence of the lower binding found in these late-stage regions (Additional file 3: Fig. S2), showing that the DVR variability with protocol non-compliance in NFT-poor regions of patients is similar to that observed on healthy brains.

A potential limitation of SUV-based quantification of [^18^F]MK-6240 is the signal spill-over from the meninges into the superior section of the cerebellum, affecting the measurements of the reference region [3, 24]. Some previous studies addressed this issue by removing the dorsal cerebellum from the reference region [3, 7, 24]; however, this decreases the signal-to-noise ratio due to its reduced size and may introduce bias on the quantification. Presently, we observed possible influence of such spill-over signal when assessing protocol delays on SUVR_90_ quantification, as longer delays in the protocol lowered the SUVR_90_ of HC subjects, showing an increased PET signal in the reference region as the delay increases.

The present work was a pilot study exploring the feasibility of the DTW protocol for [^18^F]MK-6240 studies including consideration of perfusion changes, and therefore, additional validation with larger cohorts is required. One of the current main limitations is the analysis of noiseless TACs, which does not completely represent a real scenario of PET data. Nevertheless, a similar study for amyloid tracers did not find strong effects of noise in their quantification from DTW simulations [15]. Furthermore, since the simulated TACs were extrapolated from real data of shorter acquisition time, there could be a bias on the late time point of the TAC. Another limitation is the small sample size, which makes it difficult to draw definitive conclusions on the best clinical protocol to be used for future [^18^F]MK-6240 studies; nevertheless, subjects corresponding to different levels of specific binding were evaluated. Additionally, arterial sampling failed for the two AD subjects with highest tracer binding (S7 and S8) and, therefore, their simulated TACs were generated using a population-based input curve of the other MCI/AD subjects. Their bias was already expected to be higher than lower binding subjects [4, 5], and the population-based input curve could introduce further quantitative bias. Finally, an actual dual-time-window acquisition would require alignment between the two PET acquisitions, making the analysis more laborious and potentially introduce a small bias due to image registration misalignments [28], however, this was not an issue for previously published studies using dual-time-window protocols.

## Conclusions

Reliable quantification with reference region methods for [^18^F]MK-6240 can be performed from dual-time-window PET protocols with 60-min break between two 30-min dynamic acquisitions. The Reference Logan method using the cerebellar cortex as reference region should be used as it less susceptible than SUVR estimates when perfusion changes or scanning protocol non-compliance were simulated. Moreover, the reduction of the overall acquisition time has the potential to improve patient comfort and clinical feasibility while making an efficient use of the PET imaging apparatus with interleaved scan protocols. Therefore, it can increase patient throughput and optimize tracer batch use without reducing quantitative accuracy.

## Supplementary Information

**Additional file 1.** **Fig S1:** Correlation between Reference Logan (Ref Logan) and 2 Tissue Compartment Model (2TCM) Distribution Volume Ratios (DVR), both calculated relative to the cerebellar cortex using 120 min Time Activity Curves (TACs). For the Ref Logan DVR different approaches to estimate k2’ were considered.

**Additional file 2.** **Fig S2:** Comparing Standardized Uptake Value Ratios (SUVR) using a 90 to 120 min acquisition time interval post tracer injection with Reference Logan (Ref Logan) and 2 Tissue Compartment Model (2TCM) Distribution Volume Ratios (DVR). Ratios were calculated relative to the cerebellar cortex.

**Additional file 3.** Tables with a detailed overview of the bias for the different quantification methods induced by either perfusion changes or non-compliance with the scanning protocol. **Table S1:** Bias on Reference Logan DVR due to perfusion changes with constant R1; pooling target regions. **Table S2:** Bias on Reference Logan DVR due to perfusion changes with constant R1. **Table S3:** Bias on Reference Logan DVR due to perfusion changes with variable R1; pooling target regions. **Table S4:** Bias on Reference Logan DVR due to perfusion changes with variable R1. **Table S5:** Bias on Reference Logan DVR due to implementation of the dual-time-window protocol; pooling target regions. **Table S6:** Bias on Reference Logan DVR due to implementation of the dual-time-window protocol. **Table S7:** Bias on Reference Logan DVR from DTW TAC due to perfusion changes with constant R1; pooling target regions. **Table S8:** Bias on Reference Logan DVR from DTW TAC due to perfusion changes with constant R1. **Table S9:** Bias on Reference Logan DVR from DTW TAC due to perfusion changes with variable R1; pooling target regions. **Table S10:** Bias on Reference Logan DVR from DTW TAC due to perfusion changes with variable R1. **Table S11:** Bias on Reference Logan DVR from DTW protocol non-compliance; pooling target regions. **Table S12:** Bias on Reference Logan DVR from DTW protocol non-compliance. **Table S13:** Bias on SUVR90 due to perfusion changes with constant R1; pooling target regions. **Table S14:** Bias on SUVR90 due to perfusion changes with constant R1. **Table S15:** Bias on SUVR90 due to perfusion changes with variable R1; pooling target regions. **Table S16:** Bias on SUVR90 due to perfusion changes with variable R1. **Table S17:** Bias on SUVR90 from scanning protocol non-compliance; pooling target regions. **Table S18:** Bias on SUVR90 from scanning protocol non-compliance.

## Data Availability

The datasets generated and/or analyzed during the current study are available from the corresponding author on reasonable request.

## Abbreviations

2TCM: Two-tissue compartmental model
AD: Alzheimer’s disease
AIF: Arterial input function
BBB: Blood–brain barrier
CAT: Computational Anatomy Toolbox
CBF: Cerebral blood flow
CSF: Cerebrospinal fluid
CT: Computed Tomography
DTW: Dual-time-window
DVR: Distribution volume ratio
GM: Grey matter
HC: Healthy Control
*K*_1_: Tracer influx rate from plasma to tissue
*k*_2_: Tracer clearance rate from tissue to plasma
*k*_2_’: Reference tissue clearance rate for the Reference Logan kinetic model
*k*_3_: Tracer transfer from non-displaceable to specific compartment (or transfer from free to non-specific compartment)
*k*_4_: Tracer transfer from specific to non-displaceable compartment (or from non-specific to free compartment)
MCI: Mild Cognitive Impairment
MMSE: Mini-Mental State Examination
MNI: Montreal Neurological Institute
MRI: Magnetic Resonance Imaging
NFT: Neurofibrillary tangles
PET: Positron Emission Tomography
p.i.: Post-injection
*R*_1_: Ratio between *K*_1_ of the target region and *K*_1_ of the reference region
Ref Logan: Logan reference tissue model
SD: Standard deviation
SPM: Statistical Parametric Mapping
SUVR: Standardized uptake value ratio
SUVR_90_: SUVR of the interval 90–120-min post-injection scan
TAC: Time activity curve
TAC_120_: 120-Min-long time activity curve
TAC_DTW_: Time activity curve of a dual-time-window scan
TAC_DTW40_: Time activity curve of a dual-time-window scan with 40-min-long break
TAC_DTW50_: Time activity curve of a dual-time-window scan with 50-min-long break
TAC_DTW60_: Time activity curve of a dual-time-window scan with 60-min-long break
TAC_DTW70_: Time activity curve of a dual-time-window scan with 70-min-long break
VOI: Volume of interest
*V*_T_: Total distribution volume
WM: White matter
