# Supplementary figures and images for: A dual-time-window protocol to reduce acquisition time of dynamic tau PET imaging using [18F]MK-6240
Source: EJNMMI Res. 2021 May 27;11:49. doi: 10.1186/s13550-021-00790-x (PMC8160074; doi:10.1186/s13550-021-00790-x)

# DVR Correlation

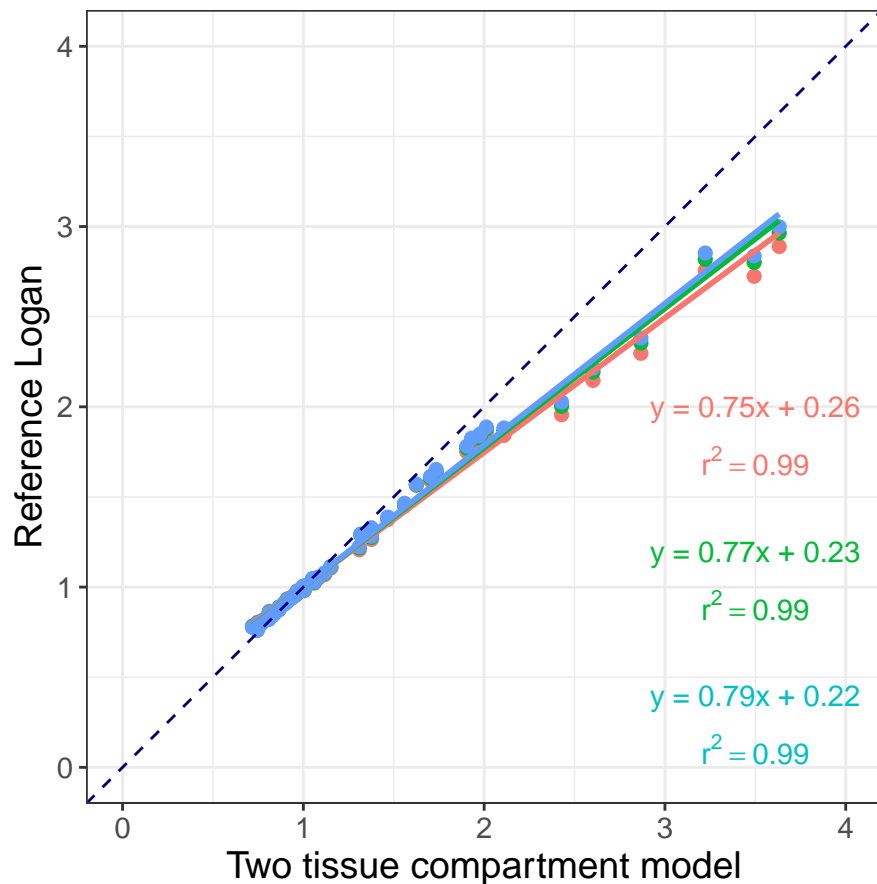

## Reference Logan k2' implementation

- Fixed k2' = 1E10
- Population k2' = 0.14386
- Individual k2' from 2TCM

Supplement: Supplementary file 1 — Additional file 1. Fig S1: Correlation between Reference Logan (Ref Logan) and 2 Tissue Compartment Model (2TCM) Distribution Volume Ratios (DVR), both calculated relative to the cerebellar cortex using 120 min Time Activity Curves (TACs). For the Ref Logan DVR different approaches to estimate k2’ were considered. [file 13550_2021_790_MOESM1_ESM.pdf]

# Comparing quantification with different models

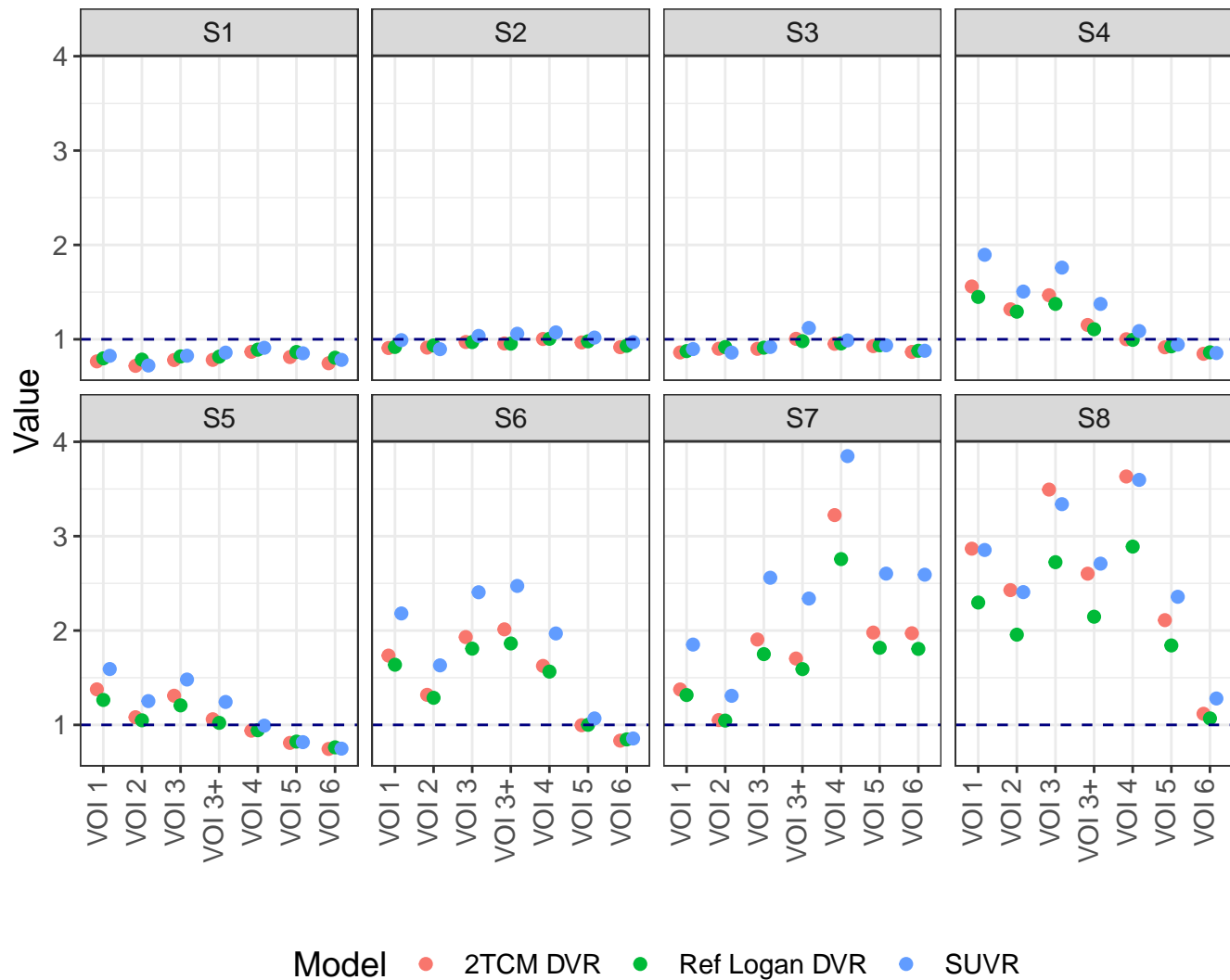

Supplement: Supplementary file 3 — Additional file 3. Tables with a detailed overview of the bias for the different quantification methods induced by either perfusion changes or non-compliance with the scanning protocol. Table S1: Bias on Reference Logan DVR due to perfusion changes with constant R1; pooling target regions. Table S2: Bias on Reference Logan DVR due to perfusion changes with constant R1. Table S3: Bias on Reference Logan DVR due to perfusion changes with variable R1; pooling target regions. Table S4: Bias on Reference Logan DVR due to perfusion changes with variable R1. Table S5: Bias on Reference Logan DVR due to implementation of the dual-time-window protocol; pooling target regions. Table S6: Bias on Reference Logan DVR due to implementation of the dual-time-window protocol. Table S7: Bias on Reference Logan DVR from DTW TAC due to perfusion changes with constant R1; pooling target regions. Table S8: Bias on Reference Logan DVR from DTW TAC due to perfusion changes with constant R1. Table S9: Bias on Reference Logan DVR from DTW TAC due to perfusion changes with variable R1; pooling target regions. Table S10: Bias on Reference Logan DVR from DTW TAC due to perfusion changes with variable R1. Table S11: Bias on Reference Logan DVR from DTW protocol non-compliance; pooling target regions. Table S12: Bias on Reference Logan DVR from DTW protocol non-compliance. Table S13: Bias on SUVR90 due to perfusion changes with constant R1; pooling target regions. Table S14: Bias on SUVR90 due to perfusion changes with constant R1. Table S15: Bias on SUVR90 due to perfusion changes with variable R1; pooling target regions. Table S16: Bias on SUVR90 due to perfusion changes with variable R1. Table S17: Bias on SUVR90 from scanning protocol non-compliance; pooling target regions. Table S18: Bias on SUVR90 from scanning protocol non-compliance. [file 13550_2021_790_MOESM3_ESM.pdf]
