# Supplementary material for: A dual-time-window protocol to reduce acquisition time of dynamic tau PET imaging using [18F]MK-6240
Source: EJNMMI Res. 2021 May 27;11:49. doi: 10.1186/s13550-021-00790-x (PMC8160074; doi:10.1186/s13550-021-00790-x)
Supplement: Supplementary file 2 — Additional file 2. Fig S2: Comparing Standardized Uptake Value Ratios (SUVR) using a 90 to 120 min acquisition time interval post tracer injection with Reference Logan (Ref Logan) and 2 Tissue Compartment Model (2TCM) Distribution Volume Ratios (DVR). Ratios were calculated relative to the cerebellar cortex. [file 13550_2021_790_MOESM2_ESM.docx]

**Supplementary Tables**

Supplementary Table 1: Bias on Reference Logan DVR due to perfusion changes with constant R1; pooling target regions

| Diagnosis | K1 change | Difference | 95% CI | | Relative difference | Relative 95% CI | | P value |
| --- | --- | --- | --- | --- | --- | --- | --- | --- |
|  |  |  | lower | upper |  | lower | upper |  |
| HC | K1-50% | -0.0004 | -0.0032 | 0.0024 | -0.04% | -0.35% | 0.27% | 0.789 |
| HC | K1-25% | 0.0003 | -0.0006 | 0.0012 | 0.03% | -0.07% | 0.13% | 0.533 |
| HC | K1-10% | 0.0001 | -0.0002 | 0.0004 | 0.01% | -0.02% | 0.05% | 0.406 |
| HC | K1+10% | -0.0001 | -0.0003 | 0.0001 | -0.02% | -0.04% | 0.01% | 0.176 |
| HC | K1+25% | -0.0003 | -0.0008 | 0.0001 | -0.04% | -0.09% | 0.02% | 0.178 |
| HC | K1+50% | -0.0006 | -0.0014 | 0.0002 | -0.07% | -0.16% | 0.02% | 0.137 |
| MCI/AD | K1-50% | -0.116 | -0.200 | -0.032 | -7.78% | -17.07% | 1.50% | 0.007 |
| MCI/AD | K1-25% | -0.037 | -0.065 | -0.008 | -2.47% | -5.61% | 0.68% | 0.011 |
| MCI/AD | K1-10% | -0.012 | -0.021 | -0.002 | -0.79% | -1.82% | 0.24% | 0.013 |
| MCI/AD | K1+10% | 0.010 | 0.002 | 0.017 | 0.65% | -0.22% | 1.52% | 0.015 |
| MCI/AD | K1+25% | 0.021 | 0.004 | 0.038 | 1.39% | -0.49% | 3.27% | 0.017 |
| MCI/AD | K1+50% | 0.033 | 0.005 | 0.061 | 2.25% | -0.86% | 5.35% | 0.019 |

Supplementary Table 2: Bias on Reference Logan DVR due to perfusion changes with constant R1

| Diagnosis | K1 change | Region | Difference | 95% CI | | Relative difference | Relative 95% CI | | P value |
| --- | --- | --- | --- | --- | --- | --- | --- | --- | --- |
|  |  |  |  | lower | upper |  | lower | upper |  |
| HC | K1-50% | VOI 1 | -0.002 | -0.003 | -0.001 | -0.26% | -0.40% | -0.13% | 0.000 |
| HC | K1-25% | VOI 1 | 0.000 | -0.001 | 0.000 | -0.03% | -0.08% | 0.01% | 0.182 |
| HC | K1-10% | VOI 1 | 0.000 | 0.000 | 0.000 | -0.01% | -0.02% | 0.01% | 0.459 |
| HC | K1+10% | VOI 1 | 0.000 | 0.000 | 0.000 | 0.00% | -0.01% | 0.01% | 0.584 |
| HC | K1+25% | VOI 1 | 0.000 | 0.000 | 0.000 | -0.01% | -0.03% | 0.02% | 0.563 |
| HC | K1+50% | VOI 1 | 0.000 | 0.000 | 0.000 | -0.02% | -0.06% | 0.02% | 0.385 |
| HC | K1-50% | VOI 2 | 0.009 | 0.005 | 0.013 | 1.00% | 0.55% | 1.44% | 0.000 |
| HC | K1-25% | VOI 2 | 0.003 | 0.002 | 0.005 | 0.39% | 0.26% | 0.52% | 0.000 |
| HC | K1-10% | VOI 2 | 0.001 | 0.001 | 0.002 | 0.14% | 0.09% | 0.18% | 0.000 |
| HC | K1+10% | VOI 2 | -0.001 | -0.001 | -0.001 | -0.11% | -0.13% | -0.08% | 0.000 |
| HC | K1+25% | VOI 2 | -0.002 | -0.003 | -0.002 | -0.24% | -0.31% | -0.18% | 0.000 |
| HC | K1+50% | VOI 2 | -0.004 | -0.005 | -0.003 | -0.41% | -0.52% | -0.30% | 0.000 |
| HC | K1-50% | VOI 3 | -0.002 | -0.006 | 0.001 | -0.24% | -0.62% | 0.14% | 0.221 |
| HC | K1-25% | VOI 3 | 0.000 | -0.001 | 0.001 | -0.03% | -0.16% | 0.10% | 0.649 |
| HC | K1-10% | VOI 3 | 0.000 | 0.000 | 0.000 | -0.01% | -0.05% | 0.04% | 0.782 |
| HC | K1+10% | VOI 3 | 0.000 | 0.000 | 0.000 | 0.00% | -0.03% | 0.03% | 0.976 |
| HC | K1+25% | VOI 3 | 0.000 | -0.001 | 0.001 | 0.00% | -0.07% | 0.07% | 0.960 |
| HC | K1+50% | VOI 3 | 0.000 | -0.001 | 0.001 | -0.01% | -0.13% | 0.11% | 0.899 |
| HC | K1-50% | VOI 3+ | -0.011 | -0.018 | -0.005 | -1.23% | -1.92% | -0.54% | 0.000 |
| HC | K1-25% | VOI 3+ | -0.003 | -0.005 | -0.001 | -0.34% | -0.57% | -0.12% | 0.003 |
| HC | K1-10% | VOI 3+ | -0.001 | -0.002 | 0.000 | -0.11% | -0.18% | -0.03% | 0.004 |
| HC | K1+10% | VOI 3+ | 0.001 | 0.000 | 0.001 | 0.08% | 0.02% | 0.14% | 0.011 |
| HC | K1+25% | VOI 3+ | 0.002 | 0.000 | 0.003 | 0.17% | 0.04% | 0.30% | 0.011 |
| HC | K1+50% | VOI 3+ | 0.003 | 0.001 | 0.005 | 0.27% | 0.06% | 0.49% | 0.013 |
| HC | K1-50% | VOI 4 | -0.003 | -0.004 | -0.002 | -0.31% | -0.43% | -0.19% | 0.000 |
| HC | K1-25% | VOI 4 | -0.001 | -0.001 | 0.000 | -0.07% | -0.11% | -0.03% | 0.000 |
| HC | K1-10% | VOI 4 | 0.000 | 0.000 | 0.000 | -0.02% | -0.03% | -0.01% | 0.001 |
| HC | K1+10% | VOI 4 | 0.000 | 0.000 | 0.000 | 0.01% | 0.00% | 0.02% | 0.014 |
| HC | K1+25% | VOI 4 | 0.000 | 0.000 | 0.000 | 0.03% | 0.01% | 0.04% | 0.014 |
| HC | K1+50% | VOI 4 | 0.000 | 0.000 | 0.001 | 0.04% | 0.01% | 0.07% | 0.022 |
| HC | K1-50% | VOI 5 | 0.003 | 0.000 | 0.007 | 0.34% | -0.02% | 0.71% | 0.066 |
| HC | K1-25% | VOI 5 | 0.001 | 0.000 | 0.002 | 0.14% | 0.01% | 0.27% | 0.031 |
| HC | K1-10% | VOI 5 | 0.000 | 0.000 | 0.001 | 0.05% | 0.00% | 0.10% | 0.033 |
| HC | K1+10% | VOI 5 | 0.000 | -0.001 | 0.000 | -0.04% | -0.07% | -0.01% | 0.007 |
| HC | K1+25% | VOI 5 | -0.001 | -0.001 | 0.000 | -0.09% | -0.16% | -0.02% | 0.010 |
| HC | K1+50% | VOI 5 | -0.001 | -0.003 | 0.000 | -0.16% | -0.28% | -0.04% | 0.007 |
| HC | K1-50% | VOI 6 | 0.004 | 0.000 | 0.008 | 0.46% | 0.00% | 0.92% | 0.050 |
| HC | K1-25% | VOI 6 | 0.002 | 0.000 | 0.003 | 0.18% | 0.02% | 0.34% | 0.024 |
| HC | K1-10% | VOI 6 | 0.001 | 0.000 | 0.001 | 0.06% | 0.01% | 0.12% | 0.025 |
| HC | K1+10% | VOI 6 | 0.000 | -0.001 | 0.000 | -0.05% | -0.09% | -0.02% | 0.006 |
| HC | K1+25% | VOI 6 | -0.001 | -0.002 | 0.000 | -0.12% | -0.20% | -0.03% | 0.008 |
| HC | K1+50% | VOI 6 | -0.002 | -0.003 | -0.001 | -0.21% | -0.35% | -0.06% | 0.005 |
| MCI/AD | K1-50% | VOI 1 | -0.135 | -0.223 | -0.046 | -8.45% | -14.02% | -2.87% | 0.003 |
| MCI/AD | K1-25% | VOI 1 | -0.043 | -0.074 | -0.012 | -2.68% | -4.62% | -0.74% | 0.007 |
| MCI/AD | K1-10% | VOI 1 | -0.014 | -0.024 | -0.003 | -0.86% | -1.50% | -0.21% | 0.009 |
| MCI/AD | K1+10% | VOI 1 | 0.011 | 0.003 | 0.020 | 0.71% | 0.17% | 1.26% | 0.010 |
| MCI/AD | K1+25% | VOI 1 | 0.024 | 0.005 | 0.043 | 1.52% | 0.34% | 2.71% | 0.012 |
| MCI/AD | K1+50% | VOI 1 | 0.039 | 0.008 | 0.071 | 2.48% | 0.51% | 4.44% | 0.013 |
| MCI/AD | K1-50% | VOI 2 | -0.078 | -0.151 | -0.004 | -5.87% | -11.40% | -0.34% | 0.038 |
| MCI/AD | K1-25% | VOI 2 | -0.024 | -0.050 | 0.001 | -1.84% | -3.75% | 0.07% | 0.060 |
| MCI/AD | K1-10% | VOI 2 | -0.008 | -0.016 | 0.001 | -0.59% | -1.23% | 0.05% | 0.070 |
| MCI/AD | K1+10% | VOI 2 | 0.006 | -0.001 | 0.013 | 0.47% | -0.05% | 0.99% | 0.075 |
| MCI/AD | K1+25% | VOI 2 | 0.013 | -0.002 | 0.029 | 1.01% | -0.13% | 2.16% | 0.082 |
| MCI/AD | K1+50% | VOI 2 | 0.022 | -0.003 | 0.047 | 1.65% | -0.25% | 3.55% | 0.089 |
| MCI/AD | K1-50% | VOI 3 | -0.179 | -0.307 | -0.051 | -10.11% | -17.35% | -2.88% | 0.006 |
| MCI/AD | K1-25% | VOI 3 | -0.057 | -0.102 | -0.013 | -3.24% | -5.77% | -0.72% | 0.012 |
| MCI/AD | K1-10% | VOI 3 | -0.019 | -0.033 | -0.004 | -1.05% | -1.89% | -0.21% | 0.015 |
| MCI/AD | K1+10% | VOI 3 | 0.015 | 0.003 | 0.028 | 0.86% | 0.15% | 1.57% | 0.018 |
| MCI/AD | K1+25% | VOI 3 | 0.033 | 0.005 | 0.060 | 1.84% | 0.29% | 3.39% | 0.020 |
| MCI/AD | K1+50% | VOI 3 | 0.053 | 0.008 | 0.099 | 2.99% | 0.43% | 5.56% | 0.022 |
| MCI/AD | K1-50% | VOI 3+ | -0.134 | -0.212 | -0.056 | -8.66% | -13.70% | -3.63% | 0.001 |
| MCI/AD | K1-25% | VOI 3+ | -0.042 | -0.067 | -0.016 | -2.69% | -4.37% | -1.01% | 0.002 |
| MCI/AD | K1-10% | VOI 3+ | -0.013 | -0.022 | -0.005 | -0.85% | -1.40% | -0.31% | 0.002 |
| MCI/AD | K1+10% | VOI 3+ | 0.011 | 0.004 | 0.018 | 0.70% | 0.23% | 1.16% | 0.003 |
| MCI/AD | K1+25% | VOI 3+ | 0.023 | 0.007 | 0.038 | 1.48% | 0.48% | 2.48% | 0.004 |
| MCI/AD | K1+50% | VOI 3+ | 0.037 | 0.011 | 0.062 | 2.39% | 0.74% | 4.03% | 0.005 |
| MCI/AD | K1-50% | VOI 4 | -0.192 | -0.371 | -0.013 | -10.51% | -20.28% | -0.73% | 0.035 |
| MCI/AD | K1-25% | VOI 4 | -0.062 | -0.121 | -0.003 | -3.39% | -6.61% | -0.16% | 0.040 |
| MCI/AD | K1-10% | VOI 4 | -0.020 | -0.039 | -0.001 | -1.09% | -2.13% | -0.04% | 0.042 |
| MCI/AD | K1+10% | VOI 4 | 0.017 | 0.001 | 0.033 | 0.91% | 0.03% | 1.78% | 0.043 |
| MCI/AD | K1+25% | VOI 4 | 0.035 | 0.001 | 0.070 | 1.93% | 0.05% | 3.82% | 0.044 |
| MCI/AD | K1+50% | VOI 4 | 0.057 | 0.001 | 0.114 | 3.13% | 0.06% | 6.21% | 0.046 |
| MCI/AD | K1-50% | VOI 5 | -0.061 | -0.125 | 0.003 | -4.75% | -9.73% | 0.23% | 0.062 |
| MCI/AD | K1-25% | VOI 5 | -0.019 | -0.039 | 0.001 | -1.48% | -3.06% | 0.10% | 0.067 |
| MCI/AD | K1-10% | VOI 5 | -0.006 | -0.013 | 0.000 | -0.47% | -0.98% | 0.04% | 0.070 |
| MCI/AD | K1+10% | VOI 5 | 0.005 | 0.000 | 0.010 | 0.39% | -0.03% | 0.81% | 0.070 |
| MCI/AD | K1+25% | VOI 5 | 0.011 | -0.001 | 0.022 | 0.82% | -0.08% | 1.72% | 0.073 |
| MCI/AD | K1+50% | VOI 5 | 0.017 | -0.002 | 0.036 | 1.32% | -0.14% | 2.79% | 0.077 |
| MCI/AD | K1-50% | VOI 6 | -0.032 | -0.080 | 0.015 | -3.03% | -7.47% | 1.41% | 0.182 |
| MCI/AD | K1-25% | VOI 6 | -0.010 | -0.024 | 0.005 | -0.93% | -2.28% | 0.43% | 0.180 |
| MCI/AD | K1-10% | VOI 6 | -0.003 | -0.008 | 0.001 | -0.29% | -0.72% | 0.14% | 0.182 |
| MCI/AD | K1+10% | VOI 6 | 0.003 | -0.001 | 0.006 | 0.24% | -0.11% | 0.58% | 0.178 |
| MCI/AD | K1+25% | VOI 6 | 0.005 | -0.003 | 0.013 | 0.50% | -0.24% | 1.23% | 0.184 |
| MCI/AD | K1+50% | VOI 6 | 0.008 | -0.004 | 0.021 | 0.78% | -0.39% | 1.96% | 0.191 |

Supplementary Table 3: Bias on Reference Logan DVR due to perfusion changes with variable R1; pooling target regions

| Diagnosis | K1 change | Difference | 95% CI | | Relative difference | Relative 95% CI | | P value |
| --- | --- | --- | --- | --- | --- | --- | --- | --- |
|  |  |  | lower | upper |  | lower | upper |  |
| HC | K1-50% | -0.011 | -0.013 | -0.009 | -1.25% | -1.47% | -1.03% | 0.000 |
| HC | K1-25% | -0.003 | -0.004 | -0.003 | -0.37% | -0.45% | -0.29% | 0.000 |
| HC | K1-10% | -0.001 | -0.001 | -0.001 | -0.12% | -0.14% | -0.09% | 0.000 |
| HC | K1+10% | 0.001 | 0.001 | 0.001 | 0.09% | 0.07% | 0.12% | 0.000 |
| HC | K1+25% | 0.002 | 0.001 | 0.002 | 0.20% | 0.14% | 0.25% | 0.000 |
| HC | K1+50% | 0.003 | 0.002 | 0.004 | 0.31% | 0.22% | 0.41% | 0.000 |
| MCI/AD | K1-50% | -0.074 | -0.127 | -0.020 | -4.94% | -8.53% | -1.35% | 0.007 |
| MCI/AD | K1-25% | -0.025 | -0.044 | -0.006 | -1.66% | -2.93% | -0.39% | 0.010 |
| MCI/AD | K1-10% | -0.008 | -0.014 | -0.002 | -0.55% | -0.97% | -0.12% | 0.012 |
| MCI/AD | K1+10% | 0.007 | 0.001 | 0.013 | 0.47% | 0.10% | 0.84% | 0.013 |
| MCI/AD | K1+25% | 0.015 | 0.003 | 0.027 | 1.02% | 0.20% | 1.83% | 0.014 |
| MCI/AD | K1+50% | 0.025 | 0.005 | 0.045 | 1.68% | 0.32% | 3.05% | 0.016 |

Supplementary Table 4: Bias on Reference Logan DVR due to perfusion changes with variable R1

| Diagnosis | K1 change | Region | Difference | 95% CI | | Relative difference | Relative 95% CI | | P value |
| --- | --- | --- | --- | --- | --- | --- | --- | --- | --- |
|  |  |  |  | lower | upper |  | lower | upper |  |
| HC | K1-50% | VOI 1 | -0.012 | -0.016 | -0.009 | -1.44% | -1.84% | -1.04% | 0.000 |
| HC | K1-25% | VOI 1 | -0.004 | -0.005 | -0.003 | -0.43% | -0.57% | -0.29% | 0.000 |
| HC | K1-10% | VOI 1 | -0.001 | -0.002 | -0.001 | -0.14% | -0.19% | -0.09% | 0.000 |
| HC | K1+10% | VOI 1 | 0.001 | 0.001 | 0.001 | 0.11% | 0.07% | 0.15% | 0.000 |
| HC | K1+25% | VOI 1 | 0.002 | 0.001 | 0.003 | 0.23% | 0.14% | 0.32% | 0.000 |
| HC | K1+50% | VOI 1 | 0.003 | 0.002 | 0.005 | 0.37% | 0.22% | 0.53% | 0.000 |
| HC | K1-50% | VOI 2 | -0.006 | -0.006 | -0.006 | -0.68% | -0.73% | -0.63% | 0.000 |
| HC | K1-25% | VOI 2 | -0.001 | -0.002 | -0.001 | -0.14% | -0.18% | -0.11% | 0.000 |
| HC | K1-10% | VOI 2 | 0.000 | 0.000 | 0.000 | -0.04% | -0.05% | -0.03% | 0.000 |
| HC | K1+10% | VOI 2 | 0.000 | 0.000 | 0.000 | 0.03% | 0.01% | 0.05% | 0.011 |
| HC | K1+25% | VOI 2 | 0.000 | 0.000 | 0.001 | 0.05% | 0.01% | 0.10% | 0.018 |
| HC | K1+50% | VOI 2 | 0.001 | 0.000 | 0.001 | 0.07% | 0.00% | 0.14% | 0.060 |
| HC | K1-50% | VOI 3 | -0.012 | -0.015 | -0.010 | -1.37% | -1.68% | -1.06% | 0.000 |
| HC | K1-25% | VOI 3 | -0.004 | -0.005 | -0.003 | -0.40% | -0.51% | -0.29% | 0.000 |
| HC | K1-10% | VOI 3 | -0.001 | -0.002 | -0.001 | -0.13% | -0.17% | -0.09% | 0.000 |
| HC | K1+10% | VOI 3 | 0.001 | 0.001 | 0.001 | 0.10% | 0.07% | 0.13% | 0.000 |
| HC | K1+25% | VOI 3 | 0.002 | 0.001 | 0.003 | 0.22% | 0.14% | 0.29% | 0.000 |
| HC | K1+50% | VOI 3 | 0.003 | 0.002 | 0.004 | 0.35% | 0.23% | 0.47% | 0.000 |
| HC | K1-50% | VOI 3+ | -0.018 | -0.019 | -0.016 | -1.95% | -2.12% | -1.78% | 0.000 |
| HC | K1-25% | VOI 3+ | -0.006 | -0.006 | -0.005 | -0.60% | -0.67% | -0.54% | 0.000 |
| HC | K1-10% | VOI 3+ | -0.002 | -0.002 | -0.002 | -0.20% | -0.22% | -0.18% | 0.000 |
| HC | K1+10% | VOI 3+ | 0.001 | 0.001 | 0.002 | 0.15% | 0.13% | 0.17% | 0.000 |
| HC | K1+25% | VOI 3+ | 0.003 | 0.003 | 0.003 | 0.33% | 0.29% | 0.38% | 0.000 |
| HC | K1+50% | VOI 3+ | 0.005 | 0.004 | 0.006 | 0.55% | 0.48% | 0.62% | 0.000 |
| HC | K1-50% | VOI 4 | -0.013 | -0.016 | -0.009 | -1.34% | -1.69% | -0.99% | 0.000 |
| HC | K1-25% | VOI 4 | -0.004 | -0.005 | -0.003 | -0.41% | -0.53% | -0.29% | 0.000 |
| HC | K1-10% | VOI 4 | -0.001 | -0.002 | -0.001 | -0.13% | -0.17% | -0.09% | 0.000 |
| HC | K1+10% | VOI 4 | 0.001 | 0.001 | 0.001 | 0.10% | 0.07% | 0.14% | 0.000 |
| HC | K1+25% | VOI 4 | 0.002 | 0.001 | 0.003 | 0.22% | 0.15% | 0.30% | 0.000 |
| HC | K1+50% | VOI 4 | 0.003 | 0.002 | 0.005 | 0.37% | 0.24% | 0.49% | 0.000 |
| HC | K1-50% | VOI 5 | -0.009 | -0.011 | -0.007 | -0.98% | -1.20% | -0.76% | 0.000 |
| HC | K1-25% | VOI 5 | -0.003 | -0.003 | -0.002 | -0.29% | -0.36% | -0.22% | 0.000 |
| HC | K1-10% | VOI 5 | -0.001 | -0.001 | -0.001 | -0.09% | -0.11% | -0.07% | 0.000 |
| HC | K1+10% | VOI 5 | 0.001 | 0.000 | 0.001 | 0.07% | 0.05% | 0.10% | 0.000 |
| HC | K1+25% | VOI 5 | 0.001 | 0.001 | 0.002 | 0.16% | 0.11% | 0.21% | 0.000 |
| HC | K1+50% | VOI 5 | 0.002 | 0.002 | 0.003 | 0.25% | 0.17% | 0.33% | 0.000 |
| HC | K1-50% | VOI 6 | -0.008 | -0.010 | -0.007 | -0.93% | -1.12% | -0.75% | 0.000 |
| HC | K1-25% | VOI 6 | -0.002 | -0.003 | -0.002 | -0.27% | -0.33% | -0.22% | 0.000 |
| HC | K1-10% | VOI 6 | -0.001 | -0.001 | -0.001 | -0.09% | -0.10% | -0.07% | 0.000 |
| HC | K1+10% | VOI 6 | 0.001 | 0.000 | 0.001 | 0.07% | 0.05% | 0.09% | 0.000 |
| HC | K1+25% | VOI 6 | 0.001 | 0.001 | 0.002 | 0.15% | 0.11% | 0.19% | 0.000 |
| HC | K1+50% | VOI 6 | 0.002 | 0.001 | 0.003 | 0.23% | 0.16% | 0.29% | 0.000 |
| MCI/AD | K1-50% | VOI 1 | -0.086 | -0.146 | -0.026 | -5.43% | -9.20% | -1.66% | 0.005 |
| MCI/AD | K1-25% | VOI 1 | -0.029 | -0.050 | -0.008 | -1.83% | -3.17% | -0.49% | 0.007 |
| MCI/AD | K1-10% | VOI 1 | -0.010 | -0.017 | -0.002 | -0.60% | -1.05% | -0.15% | 0.009 |
| MCI/AD | K1+10% | VOI 1 | 0.008 | 0.002 | 0.015 | 0.52% | 0.13% | 0.91% | 0.008 |
| MCI/AD | K1+25% | VOI 1 | 0.018 | 0.004 | 0.032 | 1.13% | 0.28% | 1.99% | 0.010 |
| MCI/AD | K1+50% | VOI 1 | 0.030 | 0.007 | 0.053 | 1.88% | 0.44% | 3.32% | 0.011 |
| MCI/AD | K1-50% | VOI 2 | -0.054 | -0.100 | -0.009 | -4.09% | -7.52% | -0.67% | 0.019 |
| MCI/AD | K1-25% | VOI 2 | -0.018 | -0.034 | -0.002 | -1.34% | -2.54% | -0.14% | 0.028 |
| MCI/AD | K1-10% | VOI 2 | -0.006 | -0.011 | 0.000 | -0.44% | -0.85% | -0.03% | 0.034 |
| MCI/AD | K1+10% | VOI 2 | 0.005 | 0.000 | 0.009 | 0.36% | 0.03% | 0.69% | 0.034 |
| MCI/AD | K1+25% | VOI 2 | 0.010 | 0.001 | 0.020 | 0.78% | 0.04% | 1.53% | 0.039 |
| MCI/AD | K1+50% | VOI 2 | 0.017 | 0.000 | 0.034 | 1.29% | 0.04% | 2.55% | 0.044 |
| MCI/AD | K1-50% | VOI 3 | -0.114 | -0.200 | -0.027 | -6.41% | -11.30% | -1.51% | 0.010 |
| MCI/AD | K1-25% | VOI 3 | -0.039 | -0.070 | -0.008 | -2.18% | -3.94% | -0.43% | 0.015 |
| MCI/AD | K1-10% | VOI 3 | -0.013 | -0.023 | -0.002 | -0.72% | -1.32% | -0.13% | 0.017 |
| MCI/AD | K1+10% | VOI 3 | 0.011 | 0.002 | 0.020 | 0.62% | 0.10% | 1.14% | 0.019 |
| MCI/AD | K1+25% | VOI 3 | 0.024 | 0.004 | 0.044 | 1.36% | 0.21% | 2.50% | 0.020 |
| MCI/AD | K1+50% | VOI 3 | 0.040 | 0.006 | 0.074 | 2.26% | 0.33% | 4.18% | 0.022 |
| MCI/AD | K1-50% | VOI 3+ | -0.084 | -0.133 | -0.035 | -5.42% | -8.59% | -2.25% | 0.001 |
| MCI/AD | K1-25% | VOI 3+ | -0.028 | -0.045 | -0.011 | -1.81% | -2.93% | -0.70% | 0.001 |
| MCI/AD | K1-10% | VOI 3+ | -0.009 | -0.015 | -0.003 | -0.60% | -0.97% | -0.22% | 0.002 |
| MCI/AD | K1+10% | VOI 3+ | 0.008 | 0.003 | 0.013 | 0.51% | 0.18% | 0.84% | 0.002 |
| MCI/AD | K1+25% | VOI 3+ | 0.017 | 0.006 | 0.028 | 1.10% | 0.39% | 1.82% | 0.002 |
| MCI/AD | K1+50% | VOI 3+ | 0.028 | 0.010 | 0.047 | 1.83% | 0.63% | 3.02% | 0.003 |
| MCI/AD | K1-50% | VOI 4 | -0.117 | -0.224 | -0.011 | -6.41% | -12.24% | -0.58% | 0.031 |
| MCI/AD | K1-25% | VOI 4 | -0.040 | -0.078 | -0.002 | -2.19% | -4.24% | -0.14% | 0.037 |
| MCI/AD | K1-10% | VOI 4 | -0.013 | -0.026 | -0.001 | -0.72% | -1.41% | -0.04% | 0.039 |
| MCI/AD | K1+10% | VOI 4 | 0.012 | 0.001 | 0.023 | 0.64% | 0.03% | 1.25% | 0.040 |
| MCI/AD | K1+25% | VOI 4 | 0.025 | 0.001 | 0.049 | 1.38% | 0.05% | 2.71% | 0.041 |
| MCI/AD | K1+50% | VOI 4 | 0.042 | 0.001 | 0.083 | 2.29% | 0.07% | 4.51% | 0.043 |
| MCI/AD | K1-50% | VOI 5 | -0.039 | -0.075 | -0.003 | -3.05% | -5.88% | -0.22% | 0.034 |
| MCI/AD | K1-25% | VOI 5 | -0.013 | -0.025 | 0.000 | -0.99% | -1.96% | -0.02% | 0.045 |
| MCI/AD | K1-10% | VOI 5 | -0.004 | -0.008 | 0.000 | -0.32% | -0.64% | 0.00% | 0.051 |
| MCI/AD | K1+10% | VOI 5 | 0.004 | 0.000 | 0.007 | 0.28% | 0.00% | 0.56% | 0.053 |
| MCI/AD | K1+25% | VOI 5 | 0.008 | 0.000 | 0.015 | 0.59% | -0.02% | 1.20% | 0.057 |
| MCI/AD | K1+50% | VOI 5 | 0.012 | -0.001 | 0.025 | 0.97% | -0.06% | 1.99% | 0.064 |
| MCI/AD | K1-50% | VOI 6 | -0.020 | -0.041 | 0.000 | -1.91% | -3.82% | 0.00% | 0.050 |
| MCI/AD | K1-25% | VOI 6 | -0.006 | -0.013 | 0.001 | -0.60% | -1.25% | 0.05% | 0.072 |
| MCI/AD | K1-10% | VOI 6 | -0.002 | -0.004 | 0.000 | -0.19% | -0.40% | 0.02% | 0.083 |
| MCI/AD | K1+10% | VOI 6 | 0.002 | 0.000 | 0.004 | 0.16% | -0.03% | 0.35% | 0.094 |
| MCI/AD | K1+25% | VOI 6 | 0.004 | -0.001 | 0.008 | 0.34% | -0.07% | 0.75% | 0.106 |
| MCI/AD | K1+50% | VOI 6 | 0.006 | -0.002 | 0.013 | 0.54% | -0.15% | 1.22% | 0.126 |

Supplementary Table 5: Bias on Reference Logan DVR due to implementation of the dual-time-window protocol; pooling target regions

| Diagnosis | Break | Difference | 95% CI | | Relative difference | Relative 95% CI | | P value |
| --- | --- | --- | --- | --- | --- | --- | --- | --- |
|  |  |  | lower | upper |  | lower | upper |  |
| HC | 70min | -0.014 | -0.024 | -0.005 | -1.61% | -2.64% | -0.58% | 0.002 |
| HC | 60min | -0.002 | -0.006 | 0.002 | -0.23% | -0.64% | 0.18% | 0.276 |
| HC | 50min | 0.000 | -0.001 | 0.001 | 0.03% | -0.10% | 0.16% | 0.663 |
| HC | 40min | 0.000 | 0.000 | 0.001 | 0.04% | 0.00% | 0.08% | 0.051 |
| MCI/AD | 70min | -0.093 | -0.134 | -0.052 | -6.24% | -8.97% | -3.51% | 0.000 |
| MCI/AD | 60min | -0.031 | -0.047 | -0.015 | -2.10% | -3.19% | -1.01% | 0.000 |
| MCI/AD | 50min | -0.012 | -0.018 | -0.005 | -0.79% | -1.24% | -0.34% | 0.001 |
| MCI/AD | 40min | -0.005 | -0.007 | -0.002 | -0.31% | -0.49% | -0.13% | 0.001 |

Supplementary Table 6: Bias on Reference Logan DVR due to implementation of the dual-time-window protocol

| Diagnosis | Break | Region | Difference | 95% CI | | Relative difference | Relative 95% CI | | P value |
| --- | --- | --- | --- | --- | --- | --- | --- | --- | --- |
|  |  |  |  | lower | upper |  | lower | upper |  |
| HC | 70min | VOI 1 | -0.021 | -0.032 | -0.010 | -2.40% | -3.65% | -1.15% | 0.000 |
| HC | 60min | VOI 1 | -0.004 | -0.009 | 0.000 | -0.49% | -1.01% | 0.03% | 0.066 |
| HC | 50min | VOI 1 | 0.000 | -0.002 | 0.001 | -0.05% | -0.23% | 0.14% | 0.613 |
| HC | 40min | VOI 1 | 0.000 | 0.000 | 0.001 | 0.02% | -0.05% | 0.08% | 0.593 |
| HC | 70min | VOI 2 | 0.004 | -0.007 | 0.014 | 0.41% | -0.78% | 1.61% | 0.497 |
| HC | 60min | VOI 2 | 0.008 | 0.003 | 0.014 | 0.95% | 0.35% | 1.54% | 0.002 |
| HC | 50min | VOI 2 | 0.005 | 0.003 | 0.007 | 0.57% | 0.31% | 0.83% | 0.000 |
| HC | 40min | VOI 2 | 0.002 | 0.001 | 0.003 | 0.26% | 0.16% | 0.37% | 0.000 |
| HC | 70min | VOI 3 | -0.008 | -0.014 | -0.002 | -0.92% | -1.60% | -0.24% | 0.008 |
| HC | 60min | VOI 3 | 0.001 | -0.002 | 0.004 | 0.13% | -0.24% | 0.50% | 0.494 |
| HC | 50min | VOI 3 | 0.001 | 0.000 | 0.003 | 0.13% | -0.04% | 0.30% | 0.132 |
| HC | 40min | VOI 3 | 0.001 | 0.000 | 0.001 | 0.06% | -0.01% | 0.13% | 0.119 |
| HC | 70min | VOI 3+ | -0.024 | -0.028 | -0.020 | -2.60% | -3.04% | -2.16% | 0.000 |
| HC | 60min | VOI 3+ | -0.005 | -0.006 | -0.004 | -0.58% | -0.69% | -0.47% | 0.000 |
| HC | 50min | VOI 3+ | -0.001 | -0.002 | -0.001 | -0.16% | -0.21% | -0.11% | 0.000 |
| HC | 40min | VOI 3+ | -0.001 | -0.001 | 0.000 | -0.06% | -0.10% | -0.02% | 0.005 |
| HC | 70min | VOI 4 | -0.014 | -0.025 | -0.003 | -1.48% | -2.60% | -0.37% | 0.009 |
| HC | 60min | VOI 4 | -0.003 | -0.008 | 0.002 | -0.34% | -0.83% | 0.16% | 0.185 |
| HC | 50min | VOI 4 | -0.001 | -0.002 | 0.001 | -0.07% | -0.23% | 0.09% | 0.404 |
| HC | 40min | VOI 4 | 0.000 | -0.001 | 0.000 | -0.01% | -0.06% | 0.03% | 0.558 |
| HC | 70min | VOI 5 | -0.018 | -0.030 | -0.006 | -1.92% | -3.23% | -0.61% | 0.004 |
| HC | 60min | VOI 5 | -0.005 | -0.010 | -0.001 | -0.57% | -1.07% | -0.08% | 0.023 |
| HC | 50min | VOI 5 | -0.001 | -0.002 | 0.000 | -0.10% | -0.24% | 0.04% | 0.180 |
| HC | 40min | VOI 5 | 0.000 | 0.000 | 0.000 | 0.00% | -0.03% | 0.04% | 0.792 |
| HC | 70min | VOI 6 | -0.020 | -0.032 | -0.009 | -2.32% | -3.62% | -1.03% | 0.000 |
| HC | 60min | VOI 6 | -0.006 | -0.010 | -0.002 | -0.68% | -1.12% | -0.24% | 0.003 |
| HC | 50min | VOI 6 | -0.001 | -0.002 | 0.000 | -0.10% | -0.22% | 0.02% | 0.088 |
| HC | 40min | VOI 6 | 0.000 | 0.000 | 0.000 | 0.01% | -0.01% | 0.04% | 0.324 |
| MCI/AD | 70min | VOI 1 | -0.110 | -0.130 | -0.091 | -6.92% | -8.14% | -5.70% | 0.000 |
| MCI/AD | 60min | VOI 1 | -0.038 | -0.046 | -0.029 | -2.36% | -2.92% | -1.81% | 0.000 |
| MCI/AD | 50min | VOI 1 | -0.015 | -0.018 | -0.011 | -0.91% | -1.16% | -0.66% | 0.000 |
| MCI/AD | 40min | VOI 1 | -0.006 | -0.007 | -0.004 | -0.36% | -0.47% | -0.26% | 0.000 |
| MCI/AD | 70min | VOI 2 | -0.075 | -0.089 | -0.061 | -5.66% | -6.74% | -4.57% | 0.000 |
| MCI/AD | 60min | VOI 2 | -0.023 | -0.028 | -0.017 | -1.71% | -2.14% | -1.27% | 0.000 |
| MCI/AD | 50min | VOI 2 | -0.008 | -0.011 | -0.005 | -0.59% | -0.80% | -0.38% | 0.000 |
| MCI/AD | 40min | VOI 2 | -0.003 | -0.004 | -0.002 | -0.22% | -0.33% | -0.12% | 0.000 |
| MCI/AD | 70min | VOI 3 | -0.119 | -0.160 | -0.078 | -6.72% | -9.02% | -4.42% | 0.000 |
| MCI/AD | 60min | VOI 3 | -0.040 | -0.057 | -0.023 | -2.25% | -3.19% | -1.32% | 0.000 |
| MCI/AD | 50min | VOI 3 | -0.016 | -0.022 | -0.009 | -0.88% | -1.27% | -0.49% | 0.000 |
| MCI/AD | 40min | VOI 3 | -0.006 | -0.009 | -0.004 | -0.36% | -0.51% | -0.20% | 0.000 |
| MCI/AD | 70min | VOI 3+ | -0.107 | -0.148 | -0.066 | -6.93% | -9.57% | -4.28% | 0.000 |
| MCI/AD | 60min | VOI 3+ | -0.036 | -0.052 | -0.019 | -2.31% | -3.37% | -1.25% | 0.000 |
| MCI/AD | 50min | VOI 3+ | -0.014 | -0.021 | -0.007 | -0.90% | -1.34% | -0.46% | 0.000 |
| MCI/AD | 40min | VOI 3+ | -0.006 | -0.008 | -0.003 | -0.37% | -0.55% | -0.19% | 0.000 |
| MCI/AD | 70min | VOI 4 | -0.112 | -0.193 | -0.031 | -6.11% | -10.53% | -1.70% | 0.007 |
| MCI/AD | 60min | VOI 4 | -0.038 | -0.068 | -0.008 | -2.08% | -3.73% | -0.44% | 0.013 |
| MCI/AD | 50min | VOI 4 | -0.015 | -0.027 | -0.003 | -0.80% | -1.46% | -0.15% | 0.016 |
| MCI/AD | 40min | VOI 4 | -0.006 | -0.011 | -0.001 | -0.32% | -0.58% | -0.06% | 0.016 |
| MCI/AD | 70min | VOI 5 | -0.070 | -0.126 | -0.014 | -5.47% | -9.86% | -1.08% | 0.015 |
| MCI/AD | 60min | VOI 5 | -0.024 | -0.047 | -0.002 | -1.91% | -3.65% | -0.16% | 0.032 |
| MCI/AD | 50min | VOI 5 | -0.009 | -0.018 | 0.001 | -0.70% | -1.43% | 0.04% | 0.064 |
| MCI/AD | 40min | VOI 5 | -0.003 | -0.007 | 0.001 | -0.26% | -0.57% | 0.04% | 0.094 |
| MCI/AD | 70min | VOI 6 | -0.057 | -0.112 | -0.002 | -5.31% | -10.48% | -0.15% | 0.044 |
| MCI/AD | 60min | VOI 6 | -0.020 | -0.043 | 0.003 | -1.88% | -3.99% | 0.24% | 0.083 |
| MCI/AD | 50min | VOI 6 | -0.007 | -0.016 | 0.003 | -0.64% | -1.54% | 0.26% | 0.163 |
| MCI/AD | 40min | VOI 6 | -0.002 | -0.006 | 0.002 | -0.23% | -0.60% | 0.15% | 0.235 |

Supplementary Table 7: Bias on Reference Logan DVR from DTW TAC due to perfusion changes with constant R1; pooling target regions

| Diagnosis | K1 change | Difference | 95% CI | | Relative difference | Relative 95% CI | | P value |
| --- | --- | --- | --- | --- | --- | --- | --- | --- |
|  |  |  | lower | upper |  | lower | upper |  |
| HC | K1-50% | -0.008 | -0.013 | -0.003 | -0.91% | -1.46% | -0.36% | 0.001 |
| HC | K1-25% | -0.003 | -0.005 | -0.001 | -0.37% | -0.57% | -0.16% | 0.000 |
| HC | K1-10% | -0.001 | -0.002 | 0.000 | -0.12% | -0.19% | -0.05% | 0.001 |
| HC | K1+10% | 0.001 | 0.000 | 0.001 | 0.09% | 0.04% | 0.14% | 0.000 |
| HC | K1+25% | 0.002 | 0.001 | 0.003 | 0.18% | 0.06% | 0.30% | 0.003 |
| HC | K1+50% | 0.002 | 0.000 | 0.004 | 0.25% | 0.05% | 0.45% | 0.015 |
| MCI/AD | K1-50% | -0.112 | -0.190 | -0.033 | -7.66% | -13.03% | -2.29% | 0.005 |
| MCI/AD | K1-25% | -0.039 | -0.066 | -0.012 | -2.65% | -4.51% | -0.80% | 0.005 |
| MCI/AD | K1-10% | -0.013 | -0.022 | -0.004 | -0.89% | -1.50% | -0.27% | 0.005 |
| MCI/AD | K1+10% | 0.011 | 0.004 | 0.019 | 0.77% | 0.24% | 1.29% | 0.004 |
| MCI/AD | K1+25% | 0.024 | 0.008 | 0.041 | 1.66% | 0.52% | 2.81% | 0.004 |
| MCI/AD | K1+50% | 0.040 | 0.012 | 0.067 | 2.73% | 0.83% | 4.63% | 0.005 |

Supplementary Table 8: Bias on Reference Logan DVR from DTW TAC due to perfusion changes with constant R1

| Diagnosis | K1 change | Region | Difference | 95% CI | | Relative difference | Relative 95% CI | | P value |
| --- | --- | --- | --- | --- | --- | --- | --- | --- | --- |
|  |  |  |  | lower | upper |  | lower | upper |  |
| HC | K1-50% | VOI 1 | -0.011 | -0.013 | -0.008 | -1.24% | -1.52% | -0.97% | 0.000 |
| HC | K1-25% | VOI 1 | -0.004 | -0.005 | -0.003 | -0.50% | -0.63% | -0.37% | 0.000 |
| HC | K1-10% | VOI 1 | -0.001 | -0.002 | -0.001 | -0.17% | -0.22% | -0.12% | 0.000 |
| HC | K1+10% | VOI 1 | 0.001 | 0.001 | 0.001 | 0.13% | 0.09% | 0.17% | 0.000 |
| HC | K1+25% | VOI 1 | 0.002 | 0.001 | 0.003 | 0.27% | 0.16% | 0.38% | 0.000 |
| HC | K1+50% | VOI 1 | 0.003 | 0.002 | 0.005 | 0.40% | 0.21% | 0.60% | 0.000 |
| HC | K1-50% | VOI 2 | -0.006 | -0.017 | 0.004 | -0.72% | -1.89% | 0.44% | 0.224 |
| HC | K1-25% | VOI 2 | -0.002 | -0.006 | 0.001 | -0.24% | -0.66% | 0.17% | 0.245 |
| HC | K1-10% | VOI 2 | -0.001 | -0.002 | 0.001 | -0.06% | -0.19% | 0.08% | 0.401 |
| HC | K1+10% | VOI 2 | 0.000 | -0.001 | 0.001 | 0.02% | -0.07% | 0.11% | 0.661 |
| HC | K1+25% | VOI 2 | 0.000 | -0.002 | 0.002 | -0.01% | -0.20% | 0.18% | 0.908 |
| HC | K1+50% | VOI 2 | -0.001 | -0.004 | 0.001 | -0.14% | -0.42% | 0.14% | 0.332 |
| HC | K1-50% | VOI 3 | -0.012 | -0.017 | -0.008 | -1.37% | -1.88% | -0.87% | 0.000 |
| HC | K1-25% | VOI 3 | -0.004 | -0.006 | -0.003 | -0.47% | -0.64% | -0.29% | 0.000 |
| HC | K1-10% | VOI 3 | -0.001 | -0.002 | -0.001 | -0.14% | -0.20% | -0.08% | 0.000 |
| HC | K1+10% | VOI 3 | 0.001 | 0.000 | 0.001 | 0.09% | 0.05% | 0.13% | 0.000 |
| HC | K1+25% | VOI 3 | 0.001 | 0.001 | 0.002 | 0.16% | 0.07% | 0.25% | 0.001 |
| HC | K1+50% | VOI 3 | 0.002 | 0.000 | 0.003 | 0.17% | 0.02% | 0.32% | 0.025 |
| HC | K1-50% | VOI 3+ | -0.020 | -0.028 | -0.013 | -2.22% | -3.05% | -1.39% | 0.000 |
| HC | K1-25% | VOI 3+ | -0.007 | -0.010 | -0.005 | -0.80% | -1.05% | -0.54% | 0.000 |
| HC | K1-10% | VOI 3+ | -0.002 | -0.003 | -0.002 | -0.26% | -0.34% | -0.18% | 0.000 |
| HC | K1+10% | VOI 3+ | 0.002 | 0.001 | 0.002 | 0.19% | 0.14% | 0.24% | 0.000 |
| HC | K1+25% | VOI 3+ | 0.004 | 0.003 | 0.004 | 0.39% | 0.29% | 0.49% | 0.000 |
| HC | K1+50% | VOI 3+ | 0.005 | 0.004 | 0.006 | 0.57% | 0.44% | 0.70% | 0.000 |
| HC | K1-50% | VOI 4 | -0.008 | -0.011 | -0.006 | -0.89% | -1.14% | -0.64% | 0.000 |
| HC | K1-25% | VOI 4 | -0.003 | -0.004 | -0.002 | -0.34% | -0.42% | -0.25% | 0.000 |
| HC | K1-10% | VOI 4 | -0.001 | -0.001 | -0.001 | -0.11% | -0.15% | -0.08% | 0.000 |
| HC | K1+10% | VOI 4 | 0.001 | 0.000 | 0.001 | 0.08% | 0.05% | 0.12% | 0.000 |
| HC | K1+25% | VOI 4 | 0.002 | 0.001 | 0.002 | 0.17% | 0.07% | 0.26% | 0.000 |
| HC | K1+50% | VOI 4 | 0.002 | 0.001 | 0.004 | 0.25% | 0.06% | 0.43% | 0.009 |
| HC | K1-50% | VOI 5 | 0.000 | -0.004 | 0.004 | 0.02% | -0.39% | 0.44% | 0.909 |
| HC | K1-25% | VOI 5 | -0.001 | -0.003 | 0.001 | -0.10% | -0.29% | 0.09% | 0.293 |
| HC | K1-10% | VOI 5 | 0.000 | -0.001 | 0.000 | -0.05% | -0.13% | 0.03% | 0.207 |
| HC | K1+10% | VOI 5 | 0.001 | 0.000 | 0.001 | 0.06% | 0.00% | 0.11% | 0.060 |
| HC | K1+25% | VOI 5 | 0.001 | 0.000 | 0.003 | 0.13% | -0.01% | 0.27% | 0.075 |
| HC | K1+50% | VOI 5 | 0.002 | 0.000 | 0.004 | 0.22% | -0.03% | 0.47% | 0.083 |
| HC | K1-50% | VOI 6 | 0.001 | -0.004 | 0.006 | 0.10% | -0.46% | 0.67% | 0.720 |
| HC | K1-25% | VOI 6 | -0.001 | -0.003 | 0.001 | -0.11% | -0.35% | 0.14% | 0.398 |
| HC | K1-10% | VOI 6 | 0.000 | -0.001 | 0.000 | -0.06% | -0.15% | 0.04% | 0.252 |
| HC | K1+10% | VOI 6 | 0.001 | 0.000 | 0.001 | 0.07% | 0.00% | 0.13% | 0.055 |
| HC | K1+25% | VOI 6 | 0.001 | 0.000 | 0.003 | 0.16% | -0.01% | 0.32% | 0.060 |
| HC | K1+50% | VOI 6 | 0.002 | 0.000 | 0.005 | 0.27% | -0.01% | 0.55% | 0.062 |
| MCI/AD | K1-50% | VOI 1 | -0.130 | -0.215 | -0.044 | -8.34% | -13.83% | -2.86% | 0.003 |
| MCI/AD | K1-25% | VOI 1 | -0.045 | -0.075 | -0.015 | -2.90% | -4.80% | -0.99% | 0.003 |
| MCI/AD | K1-10% | VOI 1 | -0.015 | -0.025 | -0.005 | -0.96% | -1.60% | -0.33% | 0.003 |
| MCI/AD | K1+10% | VOI 1 | 0.013 | 0.005 | 0.021 | 0.84% | 0.31% | 1.38% | 0.002 |
| MCI/AD | K1+25% | VOI 1 | 0.028 | 0.010 | 0.046 | 1.82% | 0.66% | 2.98% | 0.002 |
| MCI/AD | K1+50% | VOI 1 | 0.046 | 0.017 | 0.076 | 2.99% | 1.07% | 4.90% | 0.002 |
| MCI/AD | K1-50% | VOI 2 | -0.082 | -0.151 | -0.013 | -6.31% | -11.60% | -1.01% | 0.020 |
| MCI/AD | K1-25% | VOI 2 | -0.030 | -0.053 | -0.006 | -2.27% | -4.08% | -0.46% | 0.014 |
| MCI/AD | K1-10% | VOI 2 | -0.010 | -0.018 | -0.002 | -0.77% | -1.37% | -0.17% | 0.012 |
| MCI/AD | K1+10% | VOI 2 | 0.008 | 0.002 | 0.015 | 0.65% | 0.16% | 1.14% | 0.009 |
| MCI/AD | K1+25% | VOI 2 | 0.018 | 0.005 | 0.032 | 1.42% | 0.35% | 2.48% | 0.009 |
| MCI/AD | K1+50% | VOI 2 | 0.030 | 0.007 | 0.053 | 2.31% | 0.56% | 4.06% | 0.010 |
| MCI/AD | K1-50% | VOI 3 | -0.172 | -0.294 | -0.051 | -9.94% | -16.97% | -2.92% | 0.006 |
| MCI/AD | K1-25% | VOI 3 | -0.059 | -0.102 | -0.017 | -3.42% | -5.89% | -0.96% | 0.007 |
| MCI/AD | K1-10% | VOI 3 | -0.020 | -0.034 | -0.005 | -1.14% | -1.97% | -0.32% | 0.007 |
| MCI/AD | K1+10% | VOI 3 | 0.017 | 0.005 | 0.029 | 0.97% | 0.27% | 1.68% | 0.007 |
| MCI/AD | K1+25% | VOI 3 | 0.037 | 0.010 | 0.063 | 2.11% | 0.57% | 3.66% | 0.007 |
| MCI/AD | K1+50% | VOI 3 | 0.060 | 0.015 | 0.104 | 3.46% | 0.89% | 6.02% | 0.008 |
| MCI/AD | K1-50% | VOI 3+ | -0.129 | -0.201 | -0.058 | -8.57% | -13.32% | -3.83% | 0.000 |
| MCI/AD | K1-25% | VOI 3+ | -0.044 | -0.069 | -0.020 | -2.93% | -4.55% | -1.31% | 0.000 |
| MCI/AD | K1-10% | VOI 3+ | -0.015 | -0.023 | -0.007 | -0.98% | -1.51% | -0.44% | 0.000 |
| MCI/AD | K1+10% | VOI 3+ | 0.013 | 0.006 | 0.020 | 0.83% | 0.37% | 1.30% | 0.000 |
| MCI/AD | K1+25% | VOI 3+ | 0.027 | 0.012 | 0.043 | 1.80% | 0.79% | 2.82% | 0.000 |
| MCI/AD | K1+50% | VOI 3+ | 0.044 | 0.019 | 0.070 | 2.94% | 1.26% | 4.62% | 0.001 |
| MCI/AD | K1-50% | VOI 4 | -0.181 | -0.346 | -0.017 | -10.13% | -19.32% | -0.95% | 0.031 |
| MCI/AD | K1-25% | VOI 4 | -0.062 | -0.117 | -0.006 | -3.43% | -6.54% | -0.33% | 0.030 |
| MCI/AD | K1-10% | VOI 4 | -0.020 | -0.039 | -0.002 | -1.13% | -2.16% | -0.11% | 0.031 |
| MCI/AD | K1+10% | VOI 4 | 0.018 | 0.002 | 0.033 | 0.98% | 0.09% | 1.87% | 0.031 |
| MCI/AD | K1+25% | VOI 4 | 0.038 | 0.003 | 0.072 | 2.11% | 0.19% | 4.04% | 0.031 |
| MCI/AD | K1+50% | VOI 4 | 0.062 | 0.005 | 0.119 | 3.46% | 0.29% | 6.64% | 0.033 |
| MCI/AD | K1-50% | VOI 5 | -0.057 | -0.116 | 0.001 | -4.55% | -9.20% | 0.10% | 0.055 |
| MCI/AD | K1-25% | VOI 5 | -0.020 | -0.040 | -0.001 | -1.61% | -3.17% | -0.04% | 0.044 |
| MCI/AD | K1-10% | VOI 5 | -0.007 | -0.013 | 0.000 | -0.54% | -1.06% | -0.02% | 0.041 |
| MCI/AD | K1+10% | VOI 5 | 0.006 | 0.001 | 0.012 | 0.49% | 0.05% | 0.93% | 0.031 |
| MCI/AD | K1+25% | VOI 5 | 0.013 | 0.001 | 0.025 | 1.06% | 0.10% | 2.02% | 0.030 |
| MCI/AD | K1+50% | VOI 5 | 0.022 | 0.002 | 0.042 | 1.76% | 0.17% | 3.34% | 0.030 |
| MCI/AD | K1-50% | VOI 6 | -0.029 | -0.069 | 0.012 | -2.72% | -6.55% | 1.11% | 0.164 |
| MCI/AD | K1-25% | VOI 6 | -0.011 | -0.024 | 0.003 | -1.03% | -2.30% | 0.24% | 0.112 |
| MCI/AD | K1-10% | VOI 6 | -0.004 | -0.008 | 0.001 | -0.36% | -0.79% | 0.07% | 0.097 |
| MCI/AD | K1+10% | VOI 6 | 0.004 | 0.000 | 0.007 | 0.34% | -0.02% | 0.71% | 0.062 |
| MCI/AD | K1+25% | VOI 6 | 0.008 | 0.000 | 0.016 | 0.76% | -0.04% | 1.56% | 0.062 |
| MCI/AD | K1+50% | VOI 6 | 0.013 | -0.001 | 0.028 | 1.28% | -0.06% | 2.63% | 0.061 |

Supplementary Table 9: Bias on Reference Logan DVR from DTW TAC due to perfusion changes with variable R1; pooling target regions

| Diagnosis | K1 change | Difference | 95% CI | | Relative difference | Relative 95% CI | | P value |
| --- | --- | --- | --- | --- | --- | --- | --- | --- |
|  |  |  | lower | upper |  | lower | upper |  |
| HC | K1-50% | -0.010 | -0.013 | -0.008 | -1.17% | -1.41% | -0.92% | 0.000 |
| HC | K1-25% | 0.000 | -0.001 | 0.001 | 0.01% | -0.10% | 0.12% | 0.891 |
| HC | K1-10% | 0.001 | 0.000 | 0.001 | 0.06% | 0.02% | 0.10% | 0.002 |
| HC | K1+10% | -0.001 | -0.001 | -0.001 | -0.09% | -0.12% | -0.06% | 0.000 |
| HC | K1+25% | -0.002 | -0.003 | -0.002 | -0.24% | -0.31% | -0.17% | 0.000 |
| HC | K1+50% | -0.004 | -0.005 | -0.003 | -0.46% | -0.57% | -0.36% | 0.000 |
| MCI/AD | K1-50% | -0.078 | -0.138 | -0.017 | -5.30% | -9.42% | -1.18% | 0.012 |
| MCI/AD | K1-25% | -0.029 | -0.058 | -0.001 | -2.00% | -3.97% | -0.04% | 0.046 |
| MCI/AD | K1-10% | -0.014 | -0.031 | 0.003 | -0.94% | -2.11% | 0.24% | 0.117 |
| MCI/AD | K1+10% | 0.000 | -0.007 | 0.006 | -0.02% | -0.47% | 0.43% | 0.936 |
| MCI/AD | K1+25% | 0.007 | 0.003 | 0.010 | 0.45% | 0.19% | 0.71% | 0.001 |
| MCI/AD | K1+50% | 0.015 | 0.005 | 0.024 | 0.99% | 0.34% | 1.65% | 0.003 |

Supplementary Table 10: Bias on Reference Logan DVR from DTW TAC due to perfusion changes with variable R1

| Diagnosis | K1 change | Region | Difference | 95% CI | | Relative difference | Relative 95% CI | | P value |
| --- | --- | --- | --- | --- | --- | --- | --- | --- | --- |
|  |  |  |  | lower | upper |  | lower | upper |  |
| HC | K1-50% | VOI 1 | -0.013 | -0.015 | -0.011 | -1.48% | -1.73% | -1.24% | 0.000 |
| HC | K1-25% | VOI 1 | -0.001 | -0.002 | 0.000 | -0.13% | -0.25% | -0.02% | 0.022 |
| HC | K1-10% | VOI 1 | 0.000 | 0.000 | 0.000 | 0.01% | -0.03% | 0.05% | 0.643 |
| HC | K1+10% | VOI 1 | 0.000 | -0.001 | 0.000 | -0.05% | -0.08% | -0.02% | 0.004 |
| HC | K1+25% | VOI 1 | -0.001 | -0.002 | -0.001 | -0.15% | -0.21% | -0.08% | 0.000 |
| HC | K1+50% | VOI 1 | -0.003 | -0.003 | -0.002 | -0.30% | -0.39% | -0.21% | 0.000 |
| HC | K1-50% | VOI 2 | -0.012 | -0.019 | -0.005 | -1.36% | -2.19% | -0.54% | 0.001 |
| HC | K1-25% | VOI 2 | 0.000 | -0.002 | 0.003 | 0.04% | -0.26% | 0.33% | 0.810 |
| HC | K1-10% | VOI 2 | 0.001 | 0.000 | 0.002 | 0.10% | 0.00% | 0.19% | 0.044 |
| HC | K1+10% | VOI 2 | -0.001 | -0.002 | -0.001 | -0.14% | -0.20% | -0.09% | 0.000 |
| HC | K1+25% | VOI 2 | -0.004 | -0.004 | -0.003 | -0.40% | -0.50% | -0.29% | 0.000 |
| HC | K1+50% | VOI 2 | -0.007 | -0.008 | -0.006 | -0.79% | -0.92% | -0.67% | 0.000 |
| HC | K1-50% | VOI 3 | -0.014 | -0.017 | -0.012 | -1.57% | -1.84% | -1.31% | 0.000 |
| HC | K1-25% | VOI 3 | -0.001 | -0.002 | 0.001 | -0.08% | -0.24% | 0.08% | 0.324 |
| HC | K1-10% | VOI 3 | 0.000 | 0.000 | 0.001 | 0.05% | -0.01% | 0.11% | 0.128 |
| HC | K1+10% | VOI 3 | -0.001 | -0.001 | 0.000 | -0.10% | -0.15% | -0.04% | 0.000 |
| HC | K1+25% | VOI 3 | -0.002 | -0.004 | -0.001 | -0.27% | -0.39% | -0.16% | 0.000 |
| HC | K1+50% | VOI 3 | -0.005 | -0.007 | -0.003 | -0.55% | -0.74% | -0.36% | 0.000 |
| HC | K1-50% | VOI 3+ | -0.019 | -0.022 | -0.016 | -2.08% | -2.36% | -1.79% | 0.000 |
| HC | K1-25% | VOI 3+ | -0.003 | -0.004 | -0.002 | -0.33% | -0.46% | -0.20% | 0.000 |
| HC | K1-10% | VOI 3+ | 0.000 | -0.001 | 0.000 | -0.05% | -0.10% | 0.00% | 0.055 |
| HC | K1+10% | VOI 3+ | 0.000 | -0.001 | 0.000 | -0.01% | -0.06% | 0.04% | 0.661 |
| HC | K1+25% | VOI 3+ | -0.001 | -0.002 | 0.000 | -0.08% | -0.19% | 0.04% | 0.187 |
| HC | K1+50% | VOI 3+ | -0.002 | -0.004 | 0.000 | -0.21% | -0.42% | -0.01% | 0.038 |
| HC | K1-50% | VOI 4 | -0.010 | -0.013 | -0.006 | -1.01% | -1.39% | -0.63% | 0.000 |
| HC | K1-25% | VOI 4 | 0.001 | -0.001 | 0.002 | 0.07% | -0.08% | 0.23% | 0.345 |
| HC | K1-10% | VOI 4 | 0.001 | 0.000 | 0.001 | 0.08% | 0.03% | 0.13% | 0.001 |
| HC | K1+10% | VOI 4 | -0.001 | -0.001 | -0.001 | -0.11% | -0.15% | -0.07% | 0.000 |
| HC | K1+25% | VOI 4 | -0.003 | -0.003 | -0.002 | -0.27% | -0.35% | -0.19% | 0.000 |
| HC | K1+50% | VOI 4 | -0.005 | -0.006 | -0.004 | -0.49% | -0.61% | -0.38% | 0.000 |
| HC | K1-50% | VOI 5 | -0.003 | -0.004 | -0.002 | -0.34% | -0.46% | -0.21% | 0.000 |
| HC | K1-25% | VOI 5 | 0.002 | 0.002 | 0.003 | 0.25% | 0.20% | 0.30% | 0.000 |
| HC | K1-10% | VOI 5 | 0.001 | 0.001 | 0.001 | 0.13% | 0.12% | 0.14% | 0.000 |
| HC | K1+10% | VOI 5 | -0.001 | -0.001 | -0.001 | -0.12% | -0.14% | -0.10% | 0.000 |
| HC | K1+25% | VOI 5 | -0.003 | -0.003 | -0.002 | -0.28% | -0.31% | -0.26% | 0.000 |
| HC | K1+50% | VOI 5 | -0.004 | -0.005 | -0.004 | -0.48% | -0.52% | -0.44% | 0.000 |
| HC | K1-50% | VOI 6 | -0.003 | -0.003 | -0.002 | -0.31% | -0.36% | -0.26% | 0.000 |
| HC | K1-25% | VOI 6 | 0.002 | 0.002 | 0.002 | 0.23% | 0.20% | 0.26% | 0.000 |
| HC | K1-10% | VOI 6 | 0.001 | 0.001 | 0.001 | 0.11% | 0.10% | 0.13% | 0.000 |
| HC | K1+10% | VOI 6 | -0.001 | -0.001 | -0.001 | -0.11% | -0.12% | -0.09% | 0.000 |
| HC | K1+25% | VOI 6 | -0.002 | -0.002 | -0.002 | -0.25% | -0.28% | -0.22% | 0.000 |
| HC | K1+50% | VOI 6 | -0.004 | -0.004 | -0.003 | -0.42% | -0.47% | -0.37% | 0.000 |
| MCI/AD | K1-50% | VOI 1 | -0.093 | -0.166 | -0.020 | -5.96% | -10.63% | -1.29% | 0.012 |
| MCI/AD | K1-25% | VOI 1 | -0.037 | -0.074 | 0.000 | -2.38% | -4.74% | -0.01% | 0.049 |
| MCI/AD | K1-10% | VOI 1 | -0.018 | -0.042 | 0.005 | -1.18% | -2.70% | 0.34% | 0.129 |
| MCI/AD | K1+10% | VOI 1 | -0.002 | -0.013 | 0.009 | -0.13% | -0.85% | 0.59% | 0.716 |
| MCI/AD | K1+25% | VOI 1 | 0.006 | 0.002 | 0.011 | 0.41% | 0.13% | 0.68% | 0.004 |
| MCI/AD | K1+50% | VOI 1 | 0.016 | 0.011 | 0.021 | 1.04% | 0.73% | 1.35% | 0.000 |
| MCI/AD | K1-50% | VOI 2 | -0.076 | -0.146 | -0.006 | -5.74% | -11.04% | -0.44% | 0.034 |
| MCI/AD | K1-25% | VOI 2 | -0.036 | -0.079 | 0.008 | -2.70% | -6.00% | 0.60% | 0.109 |
| MCI/AD | K1-10% | VOI 2 | -0.023 | -0.057 | 0.011 | -1.72% | -4.31% | 0.87% | 0.193 |
| MCI/AD | K1+10% | VOI 2 | -0.012 | -0.037 | 0.013 | -0.91% | -2.83% | 1.02% | 0.356 |
| MCI/AD | K1+25% | VOI 2 | -0.007 | -0.027 | 0.014 | -0.51% | -2.08% | 1.05% | 0.519 |
| MCI/AD | K1+50% | VOI 2 | -0.001 | -0.016 | 0.014 | -0.09% | -1.20% | 1.03% | 0.877 |
| MCI/AD | K1-50% | VOI 3 | -0.119 | -0.216 | -0.021 | -6.81% | -12.39% | -1.23% | 0.017 |
| MCI/AD | K1-25% | VOI 3 | -0.046 | -0.092 | 0.000 | -2.64% | -5.26% | -0.02% | 0.048 |
| MCI/AD | K1-10% | VOI 3 | -0.021 | -0.047 | 0.005 | -1.22% | -2.72% | 0.29% | 0.113 |
| MCI/AD | K1+10% | VOI 3 | 0.001 | -0.007 | 0.009 | 0.05% | -0.40% | 0.50% | 0.833 |
| MCI/AD | K1+25% | VOI 3 | 0.012 | 0.008 | 0.017 | 0.71% | 0.46% | 0.95% | 0.000 |
| MCI/AD | K1+50% | VOI 3 | 0.026 | 0.009 | 0.043 | 1.48% | 0.51% | 2.45% | 0.003 |
| MCI/AD | K1-50% | VOI 3+ | -0.086 | -0.138 | -0.034 | -5.68% | -9.10% | -2.26% | 0.001 |
| MCI/AD | K1-25% | VOI 3+ | -0.031 | -0.054 | -0.008 | -2.05% | -3.57% | -0.54% | 0.008 |
| MCI/AD | K1-10% | VOI 3+ | -0.013 | -0.025 | 0.000 | -0.84% | -1.66% | -0.02% | 0.043 |
| MCI/AD | K1+10% | VOI 3+ | 0.003 | 0.000 | 0.007 | 0.22% | 0.00% | 0.44% | 0.051 |
| MCI/AD | K1+25% | VOI 3+ | 0.012 | 0.007 | 0.016 | 0.76% | 0.43% | 1.09% | 0.000 |
| MCI/AD | K1+50% | VOI 3+ | 0.021 | 0.009 | 0.033 | 1.38% | 0.61% | 2.16% | 0.000 |
| MCI/AD | K1-50% | VOI 4 | -0.113 | -0.214 | -0.011 | -6.28% | -11.92% | -0.63% | 0.029 |
| MCI/AD | K1-25% | VOI 4 | -0.039 | -0.078 | 0.001 | -2.17% | -4.37% | 0.03% | 0.053 |
| MCI/AD | K1-10% | VOI 4 | -0.015 | -0.031 | 0.002 | -0.81% | -1.73% | 0.11% | 0.083 |
| MCI/AD | K1+10% | VOI 4 | 0.007 | 0.000 | 0.014 | 0.39% | 0.00% | 0.78% | 0.050 |
| MCI/AD | K1+25% | VOI 4 | 0.018 | -0.001 | 0.037 | 1.02% | -0.05% | 2.09% | 0.061 |
| MCI/AD | K1+50% | VOI 4 | 0.032 | -0.003 | 0.067 | 1.77% | -0.17% | 3.71% | 0.074 |
| MCI/AD | K1-50% | VOI 5 | -0.038 | -0.074 | -0.002 | -3.03% | -5.88% | -0.19% | 0.037 |
| MCI/AD | K1-25% | VOI 5 | -0.012 | -0.026 | 0.003 | -0.93% | -2.08% | 0.22% | 0.112 |
| MCI/AD | K1-10% | VOI 5 | -0.005 | -0.011 | 0.002 | -0.36% | -0.89% | 0.16% | 0.175 |
| MCI/AD | K1+10% | VOI 5 | 0.001 | -0.001 | 0.003 | 0.07% | -0.07% | 0.22% | 0.334 |
| MCI/AD | K1+25% | VOI 5 | 0.003 | -0.002 | 0.009 | 0.28% | -0.15% | 0.70% | 0.202 |
| MCI/AD | K1+50% | VOI 5 | 0.006 | -0.004 | 0.017 | 0.51% | -0.31% | 1.33% | 0.221 |
| MCI/AD | K1-50% | VOI 6 | -0.018 | -0.036 | -0.001 | -1.74% | -3.42% | -0.05% | 0.044 |
| MCI/AD | K1-25% | VOI 6 | -0.005 | -0.011 | 0.002 | -0.44% | -1.08% | 0.19% | 0.172 |
| MCI/AD | K1-10% | VOI 6 | -0.002 | -0.005 | 0.001 | -0.17% | -0.43% | 0.09% | 0.208 |
| MCI/AD | K1+10% | VOI 6 | 0.000 | -0.002 | 0.002 | 0.01% | -0.19% | 0.21% | 0.932 |
| MCI/AD | K1+25% | VOI 6 | 0.001 | -0.003 | 0.005 | 0.08% | -0.29% | 0.46% | 0.664 |
| MCI/AD | K1+50% | VOI 6 | 0.002 | -0.005 | 0.008 | 0.16% | -0.45% | 0.78% | 0.605 |

Supplementary Table 11: Bias on Reference Logan DVR from DTW protocol non-compliance; pooling target regions

| Diagnosis | Delay | Difference | 95% CI | | Relative difference | Relative 95% CI | | P value |
| --- | --- | --- | --- | --- | --- | --- | --- | --- |
|  |  |  | lower | upper |  | lower | upper |  |
| HC | 5min | -0.001 | -0.002 | 0.000 | -0.10% | -0.19% | -0.01% | 0.036 |
| HC | 10min | -0.002 | -0.003 | 0.000 | -0.19% | -0.38% | 0.00% | 0.047 |
| HC | 20min | -0.003 | -0.006 | 0.000 | -0.33% | -0.70% | 0.03% | 0.073 |
| MCI/AD | 5min | 0.005 | -0.002 | 0.012 | 0.35% | -0.12% | 0.82% | 0.140 |
| MCI/AD | 10min | 0.009 | -0.004 | 0.022 | 0.61% | -0.28% | 1.50% | 0.178 |
| MCI/AD | 20min | 0.013 | -0.010 | 0.037 | 0.92% | -0.68% | 2.52% | 0.261 |

Supplementary Table 12: Bias on Reference Logan DVR from DTW protocol non-compliance

| Diagnosis | Delay | Region | Difference | 95% CI | | Relative difference | Relative 95% CI | | P value |
| --- | --- | --- | --- | --- | --- | --- | --- | --- | --- |
|  |  |  |  | lower | upper |  | lower | upper |  |
| HC | 5min | VOI 1 | -0.001 | -0.001 | -0.001 | -0.12% | -0.17% | -0.06% | 0.000 |
| HC | 10min | VOI 1 | -0.002 | -0.003 | -0.001 | -0.22% | -0.33% | -0.11% | 0.000 |
| HC | 20min | VOI 1 | -0.003 | -0.005 | -0.001 | -0.38% | -0.61% | -0.15% | 0.001 |
| HC | 5min | VOI 2 | -0.001 | -0.002 | 0.000 | -0.11% | -0.27% | 0.05% | 0.165 |
| HC | 10min | VOI 2 | -0.002 | -0.005 | 0.001 | -0.19% | -0.52% | 0.13% | 0.242 |
| HC | 20min | VOI 2 | -0.002 | -0.008 | 0.003 | -0.26% | -0.90% | 0.38% | 0.430 |
| HC | 5min | VOI 3 | 0.000 | -0.001 | 0.000 | -0.04% | -0.12% | 0.04% | 0.366 |
| HC | 10min | VOI 3 | -0.001 | -0.002 | 0.001 | -0.07% | -0.22% | 0.09% | 0.418 |
| HC | 20min | VOI 3 | -0.001 | -0.004 | 0.002 | -0.10% | -0.40% | 0.21% | 0.526 |
| HC | 5min | VOI 3+ | 0.000 | -0.001 | 0.001 | -0.02% | -0.15% | 0.12% | 0.789 |
| HC | 10min | VOI 3+ | 0.000 | -0.003 | 0.002 | -0.05% | -0.30% | 0.21% | 0.731 |
| HC | 20min | VOI 3+ | -0.001 | -0.005 | 0.003 | -0.12% | -0.57% | 0.33% | 0.604 |
| HC | 5min | VOI 4 | -0.001 | -0.001 | 0.000 | -0.06% | -0.11% | -0.01% | 0.022 |
| HC | 10min | VOI 4 | -0.001 | -0.002 | 0.000 | -0.11% | -0.21% | -0.01% | 0.027 |
| HC | 20min | VOI 4 | -0.002 | -0.004 | 0.000 | -0.20% | -0.39% | -0.01% | 0.037 |
| HC | 5min | VOI 5 | -0.002 | -0.003 | -0.001 | -0.17% | -0.27% | -0.07% | 0.001 |
| HC | 10min | VOI 5 | -0.003 | -0.005 | -0.001 | -0.32% | -0.53% | -0.12% | 0.002 |
| HC | 20min | VOI 5 | -0.005 | -0.009 | -0.002 | -0.59% | -0.98% | -0.20% | 0.003 |
| HC | 5min | VOI 6 | -0.002 | -0.003 | -0.001 | -0.20% | -0.30% | -0.10% | 0.000 |
| HC | 10min | VOI 6 | -0.003 | -0.005 | -0.002 | -0.38% | -0.59% | -0.17% | 0.000 |
| HC | 20min | VOI 6 | -0.006 | -0.009 | -0.003 | -0.69% | -1.09% | -0.29% | 0.001 |
| MCI/AD | 5min | VOI 1 | 0.006 | -0.002 | 0.015 | 0.40% | -0.14% | 0.94% | 0.151 |
| MCI/AD | 10min | VOI 1 | 0.011 | -0.005 | 0.027 | 0.69% | -0.34% | 1.72% | 0.194 |
| MCI/AD | 20min | VOI 1 | 0.016 | -0.013 | 0.045 | 1.03% | -0.85% | 2.92% | 0.284 |
| MCI/AD | 5min | VOI 2 | 0.004 | -0.004 | 0.011 | 0.30% | -0.28% | 0.88% | 0.309 |
| MCI/AD | 10min | VOI 2 | 0.007 | -0.007 | 0.021 | 0.54% | -0.56% | 1.64% | 0.337 |
| MCI/AD | 20min | VOI 2 | 0.011 | -0.014 | 0.037 | 0.88% | -1.09% | 2.85% | 0.384 |
| MCI/AD | 5min | VOI 3 | 0.009 | -0.002 | 0.020 | 0.50% | -0.14% | 1.14% | 0.129 |
| MCI/AD | 10min | VOI 3 | 0.015 | -0.006 | 0.037 | 0.89% | -0.34% | 2.11% | 0.159 |
| MCI/AD | 20min | VOI 3 | 0.024 | -0.015 | 0.063 | 1.40% | -0.85% | 3.65% | 0.226 |
| MCI/AD | 5min | VOI 3+ | 0.005 | -0.001 | 0.011 | 0.33% | -0.09% | 0.76% | 0.129 |
| MCI/AD | 10min | VOI 3+ | 0.008 | -0.004 | 0.021 | 0.56% | -0.25% | 1.36% | 0.176 |
| MCI/AD | 20min | VOI 3+ | 0.012 | -0.010 | 0.034 | 0.79% | -0.68% | 2.25% | 0.295 |
| MCI/AD | 5min | VOI 4 | 0.010 | -0.002 | 0.021 | 0.53% | -0.10% | 1.17% | 0.102 |
| MCI/AD | 10min | VOI 4 | 0.017 | -0.004 | 0.038 | 0.94% | -0.23% | 2.12% | 0.118 |
| MCI/AD | 20min | VOI 4 | 0.027 | -0.010 | 0.063 | 1.49% | -0.55% | 3.53% | 0.156 |
| MCI/AD | 5min | VOI 5 | 0.002 | -0.002 | 0.006 | 0.17% | -0.12% | 0.47% | 0.250 |
| MCI/AD | 10min | VOI 5 | 0.003 | -0.003 | 0.010 | 0.27% | -0.26% | 0.81% | 0.316 |
| MCI/AD | 20min | VOI 5 | 0.004 | -0.007 | 0.016 | 0.33% | -0.58% | 1.24% | 0.477 |
| MCI/AD | 5min | VOI 6 | 0.000 | 0.000 | 0.001 | 0.04% | -0.04% | 0.13% | 0.314 |
| MCI/AD | 10min | VOI 6 | 0.000 | -0.001 | 0.002 | 0.03% | -0.08% | 0.15% | 0.586 |
| MCI/AD | 20min | VOI 6 | -0.001 | -0.003 | 0.001 | -0.09% | -0.29% | 0.11% | 0.378 |

Supplementary Table 13: Bias on SUVR_90_ due to perfusion changes with constant R1; pooling target regions

| Diagnosis | K1 change | Difference | 95% CI | | Relative difference | Relative 95% CI | | P value |
| --- | --- | --- | --- | --- | --- | --- | --- | --- |
|  |  |  | lower | upper |  | lower | upper |  |
| HC | K1-50% | 0.016 | 0.012 | 0.021 | 1.76% | 1.29% | 2.23% | 0.000 |
| HC | K1-25% | 0.007 | 0.006 | 0.009 | 0.78% | 0.62% | 0.94% | 0.000 |
| HC | K1-10% | 0.003 | 0.002 | 0.003 | 0.28% | 0.22% | 0.33% | 0.000 |
| HC | K1+10% | -0.002 | -0.003 | -0.002 | -0.24% | -0.28% | -0.19% | 0.000 |
| HC | K1+25% | -0.005 | -0.006 | -0.004 | -0.54% | -0.63% | -0.44% | 0.000 |
| HC | K1+50% | -0.008 | -0.010 | -0.007 | -0.92% | -1.08% | -0.76% | 0.000 |
| MCI/AD | K1-50% | -0.283 | -0.471 | -0.095 | -15.06% | -25.06% | -5.07% | 0.003 |
| MCI/AD | K1-25% | -0.095 | -0.166 | -0.025 | -5.08% | -8.84% | -1.31% | 0.008 |
| MCI/AD | K1-10% | -0.030 | -0.055 | -0.006 | -1.60% | -2.90% | -0.30% | 0.016 |
| MCI/AD | K1+10% | 0.027 | 0.007 | 0.048 | 1.46% | 0.37% | 2.54% | 0.008 |
| MCI/AD | K1+25% | 0.057 | 0.012 | 0.102 | 3.02% | 0.62% | 5.42% | 0.014 |
| MCI/AD | K1+50% | 0.090 | 0.015 | 0.166 | 4.81% | 0.78% | 8.84% | 0.019 |

Supplementary Table 14: Bias on SUVR_90_ due to perfusion changes with constant R1

| Diagnosis | K1 change | Region | Difference | 95% CI | | Relative difference | Relative 95% CI | | P value |
| --- | --- | --- | --- | --- | --- | --- | --- | --- | --- |
|  |  |  |  | lower | upper |  | lower | upper |  |
| HC | K1-50% | VOI 1 | 0.016 | 0.003 | 0.028 | 1.73% | 0.35% | 3.12% | 0.014 |
| HC | K1-25% | VOI 1 | 0.008 | 0.003 | 0.013 | 0.86% | 0.33% | 1.38% | 0.001 |
| HC | K1-10% | VOI 1 | 0.003 | 0.001 | 0.004 | 0.32% | 0.14% | 0.49% | 0.001 |
| HC | K1+10% | VOI 1 | -0.002 | -0.004 | -0.001 | -0.27% | -0.43% | -0.11% | 0.001 |
| HC | K1+25% | VOI 1 | -0.006 | -0.009 | -0.002 | -0.62% | -0.96% | -0.28% | 0.000 |
| HC | K1+50% | VOI 1 | -0.010 | -0.015 | -0.005 | -1.07% | -1.64% | -0.50% | 0.000 |
| HC | K1-50% | VOI 2 | 0.056 | 0.043 | 0.069 | 6.80% | 5.23% | 8.37% | 0.000 |
| HC | K1-25% | VOI 2 | 0.020 | 0.015 | 0.025 | 2.45% | 1.83% | 3.08% | 0.000 |
| HC | K1-10% | VOI 2 | 0.007 | 0.005 | 0.008 | 0.81% | 0.60% | 1.03% | 0.000 |
| HC | K1+10% | VOI 2 | -0.006 | -0.007 | -0.004 | -0.69% | -0.86% | -0.53% | 0.000 |
| HC | K1+25% | VOI 2 | -0.012 | -0.015 | -0.009 | -1.50% | -1.86% | -1.14% | 0.000 |
| HC | K1+50% | VOI 2 | -0.020 | -0.025 | -0.015 | -2.47% | -3.06% | -1.88% | 0.000 |
| HC | K1-50% | VOI 3 | 0.019 | 0.003 | 0.035 | 2.06% | 0.32% | 3.80% | 0.020 |
| HC | K1-25% | VOI 3 | 0.008 | 0.002 | 0.014 | 0.90% | 0.26% | 1.54% | 0.006 |
| HC | K1-10% | VOI 3 | 0.003 | 0.001 | 0.005 | 0.32% | 0.11% | 0.54% | 0.003 |
| HC | K1+10% | VOI 3 | -0.003 | -0.004 | -0.001 | -0.27% | -0.46% | -0.09% | 0.003 |
| HC | K1+25% | VOI 3 | -0.006 | -0.009 | -0.002 | -0.62% | -1.01% | -0.22% | 0.002 |
| HC | K1+50% | VOI 3 | -0.010 | -0.016 | -0.004 | -1.05% | -1.71% | -0.40% | 0.002 |
| HC | K1-50% | VOI 3+ | -0.007 | -0.021 | 0.006 | -0.73% | -2.10% | 0.64% | 0.295 |
| HC | K1-25% | VOI 3+ | 0.000 | -0.004 | 0.005 | 0.04% | -0.39% | 0.47% | 0.857 |
| HC | K1-10% | VOI 3+ | 0.001 | -0.001 | 0.002 | 0.05% | -0.09% | 0.19% | 0.489 |
| HC | K1+10% | VOI 3+ | -0.001 | -0.002 | 0.001 | -0.05% | -0.15% | 0.05% | 0.330 |
| HC | K1+25% | VOI 3+ | -0.002 | -0.004 | 0.001 | -0.15% | -0.38% | 0.08% | 0.194 |
| HC | K1+50% | VOI 3+ | -0.003 | -0.007 | 0.001 | -0.31% | -0.68% | 0.05% | 0.095 |
| HC | K1-50% | VOI 4 | 0.005 | -0.003 | 0.013 | 0.54% | -0.29% | 1.36% | 0.201 |
| HC | K1-25% | VOI 4 | 0.003 | 0.000 | 0.006 | 0.33% | 0.04% | 0.61% | 0.023 |
| HC | K1-10% | VOI 4 | 0.001 | 0.000 | 0.002 | 0.13% | 0.03% | 0.22% | 0.009 |
| HC | K1+10% | VOI 4 | -0.001 | -0.002 | 0.000 | -0.11% | -0.19% | -0.03% | 0.008 |
| HC | K1+25% | VOI 4 | -0.003 | -0.004 | -0.001 | -0.25% | -0.43% | -0.08% | 0.004 |
| HC | K1+50% | VOI 4 | -0.004 | -0.007 | -0.002 | -0.45% | -0.74% | -0.16% | 0.002 |
| HC | K1-50% | VOI 5 | 0.012 | 0.005 | 0.018 | 1.27% | 0.58% | 1.96% | 0.000 |
| HC | K1-25% | VOI 5 | 0.005 | 0.003 | 0.007 | 0.54% | 0.33% | 0.74% | 0.000 |
| HC | K1-10% | VOI 5 | 0.002 | 0.001 | 0.002 | 0.19% | 0.12% | 0.25% | 0.000 |
| HC | K1+10% | VOI 5 | -0.002 | -0.002 | -0.001 | -0.16% | -0.21% | -0.11% | 0.000 |
| HC | K1+25% | VOI 5 | -0.003 | -0.004 | -0.002 | -0.36% | -0.47% | -0.25% | 0.000 |
| HC | K1+50% | VOI 5 | -0.006 | -0.007 | -0.004 | -0.62% | -0.80% | -0.43% | 0.000 |
| HC | K1-50% | VOI 6 | 0.013 | 0.006 | 0.020 | 1.50% | 0.72% | 2.29% | 0.000 |
| HC | K1-25% | VOI 6 | 0.005 | 0.003 | 0.008 | 0.62% | 0.37% | 0.88% | 0.000 |
| HC | K1-10% | VOI 6 | 0.002 | 0.001 | 0.003 | 0.22% | 0.14% | 0.30% | 0.000 |
| HC | K1+10% | VOI 6 | -0.002 | -0.002 | -0.001 | -0.19% | -0.26% | -0.12% | 0.000 |
| HC | K1+25% | VOI 6 | -0.004 | -0.005 | -0.002 | -0.42% | -0.57% | -0.27% | 0.000 |
| HC | K1+50% | VOI 6 | -0.006 | -0.008 | -0.004 | -0.71% | -0.97% | -0.46% | 0.000 |
| MCI/AD | K1-50% | VOI 1 | -0.329 | -0.487 | -0.171 | -15.86% | -23.45% | -8.26% | 0.000 |
| MCI/AD | K1-25% | VOI 1 | -0.109 | -0.172 | -0.045 | -5.24% | -8.30% | -2.19% | 0.001 |
| MCI/AD | K1-10% | VOI 1 | -0.034 | -0.056 | -0.011 | -1.63% | -2.71% | -0.54% | 0.003 |
| MCI/AD | K1+10% | VOI 1 | 0.032 | 0.012 | 0.052 | 1.53% | 0.56% | 2.49% | 0.002 |
| MCI/AD | K1+25% | VOI 1 | 0.065 | 0.020 | 0.109 | 3.13% | 0.98% | 5.28% | 0.004 |
| MCI/AD | K1+50% | VOI 1 | 0.103 | 0.026 | 0.179 | 4.94% | 1.26% | 8.63% | 0.009 |
| MCI/AD | K1-50% | VOI 2 | -0.155 | -0.303 | -0.008 | -9.59% | -18.69% | -0.50% | 0.039 |
| MCI/AD | K1-25% | VOI 2 | -0.044 | -0.103 | 0.014 | -2.72% | -6.33% | 0.89% | 0.140 |
| MCI/AD | K1-10% | VOI 2 | -0.012 | -0.033 | 0.008 | -0.75% | -2.02% | 0.51% | 0.244 |
| MCI/AD | K1+10% | VOI 2 | 0.012 | -0.006 | 0.030 | 0.75% | -0.38% | 1.88% | 0.191 |
| MCI/AD | K1+25% | VOI 2 | 0.023 | -0.018 | 0.064 | 1.42% | -1.09% | 3.93% | 0.267 |
| MCI/AD | K1+50% | VOI 2 | 0.034 | -0.036 | 0.103 | 2.09% | -2.19% | 6.37% | 0.339 |
| MCI/AD | K1-50% | VOI 3 | -0.425 | -0.666 | -0.184 | -18.40% | -28.84% | -7.97% | 0.001 |
| MCI/AD | K1-25% | VOI 3 | -0.145 | -0.241 | -0.050 | -6.29% | -10.43% | -2.16% | 0.003 |
| MCI/AD | K1-10% | VOI 3 | -0.046 | -0.080 | -0.012 | -2.00% | -3.46% | -0.54% | 0.007 |
| MCI/AD | K1+10% | VOI 3 | 0.042 | 0.013 | 0.071 | 1.83% | 0.57% | 3.08% | 0.004 |
| MCI/AD | K1+25% | VOI 3 | 0.088 | 0.023 | 0.153 | 3.80% | 0.99% | 6.61% | 0.008 |
| MCI/AD | K1+50% | VOI 3 | 0.140 | 0.029 | 0.251 | 6.07% | 1.26% | 10.87% | 0.013 |
| MCI/AD | K1-50% | VOI 3+ | -0.338 | -0.522 | -0.154 | -16.69% | -25.76% | -7.62% | 0.000 |
| MCI/AD | K1-25% | VOI 3+ | -0.110 | -0.177 | -0.043 | -5.43% | -8.72% | -2.14% | 0.001 |
| MCI/AD | K1-10% | VOI 3+ | -0.034 | -0.056 | -0.011 | -1.66% | -2.76% | -0.56% | 0.003 |
| MCI/AD | K1+10% | VOI 3+ | 0.031 | 0.011 | 0.051 | 1.52% | 0.55% | 2.50% | 0.002 |
| MCI/AD | K1+25% | VOI 3+ | 0.062 | 0.020 | 0.104 | 3.06% | 0.97% | 5.15% | 0.004 |
| MCI/AD | K1+50% | VOI 3+ | 0.096 | 0.025 | 0.166 | 4.71% | 1.25% | 8.17% | 0.008 |
| MCI/AD | K1-50% | VOI 4 | -0.445 | -0.866 | -0.024 | -19.36% | -37.69% | -1.04% | 0.038 |
| MCI/AD | K1-25% | VOI 4 | -0.162 | -0.324 | 0.000 | -7.04% | -14.09% | 0.00% | 0.050 |
| MCI/AD | K1-10% | VOI 4 | -0.054 | -0.110 | 0.002 | -2.34% | -4.78% | 0.11% | 0.061 |
| MCI/AD | K1+10% | VOI 4 | 0.048 | 0.001 | 0.095 | 2.08% | 0.05% | 4.11% | 0.045 |
| MCI/AD | K1+25% | VOI 4 | 0.103 | -0.001 | 0.207 | 4.47% | -0.04% | 8.99% | 0.052 |
| MCI/AD | K1+50% | VOI 4 | 0.169 | -0.005 | 0.343 | 7.35% | -0.22% | 14.91% | 0.057 |
| MCI/AD | K1-50% | VOI 5 | -0.173 | -0.361 | 0.014 | -11.14% | -23.18% | 0.91% | 0.070 |
| MCI/AD | K1-25% | VOI 5 | -0.058 | -0.124 | 0.007 | -3.75% | -7.95% | 0.44% | 0.079 |
| MCI/AD | K1-10% | VOI 5 | -0.019 | -0.040 | 0.003 | -1.21% | -2.59% | 0.18% | 0.089 |
| MCI/AD | K1+10% | VOI 5 | 0.016 | -0.002 | 0.034 | 1.04% | -0.10% | 2.18% | 0.074 |
| MCI/AD | K1+25% | VOI 5 | 0.034 | -0.004 | 0.073 | 2.19% | -0.27% | 4.66% | 0.081 |
| MCI/AD | K1+50% | VOI 5 | 0.055 | -0.008 | 0.118 | 3.52% | -0.50% | 7.55% | 0.086 |
| MCI/AD | K1-50% | VOI 6 | -0.115 | -0.279 | 0.049 | -9.10% | -22.07% | 3.86% | 0.169 |
| MCI/AD | K1-25% | VOI 6 | -0.039 | -0.096 | 0.018 | -3.11% | -7.62% | 1.39% | 0.175 |
| MCI/AD | K1-10% | VOI 6 | -0.013 | -0.032 | 0.006 | -1.02% | -2.52% | 0.48% | 0.183 |
| MCI/AD | K1+10% | VOI 6 | 0.011 | -0.004 | 0.026 | 0.84% | -0.35% | 2.03% | 0.166 |
| MCI/AD | K1+25% | VOI 6 | 0.023 | -0.010 | 0.056 | 1.80% | -0.79% | 4.39% | 0.172 |
| MCI/AD | K1+50% | VOI 6 | 0.037 | -0.016 | 0.090 | 2.91% | -1.30% | 7.13% | 0.175 |

Supplementary Table 15: Bias on SUVR_90_ due to perfusion changes with variable R1; pooling target regions

| Diagnosis | K1 change | Difference | 95% CI | | Relative difference | Relative 95% CI | | P value |
| --- | --- | --- | --- | --- | --- | --- | --- | --- |
|  |  |  | lower | upper |  | lower | upper |  |
| HC | K1-50% | 0.087 | 0.054 | 0.120 | 9.41% | 5.87% | 12.96% | 0.000 |
| HC | K1-25% | 0.031 | 0.020 | 0.042 | 3.35% | 2.14% | 4.57% | 0.000 |
| HC | K1-10% | 0.010 | 0.007 | 0.014 | 1.14% | 0.73% | 1.54% | 0.000 |
| HC | K1+10% | -0.009 | -0.012 | -0.006 | -0.94% | -1.27% | -0.61% | 0.000 |
| HC | K1+25% | -0.019 | -0.026 | -0.013 | -2.08% | -2.80% | -1.35% | 0.000 |
| HC | K1+50% | -0.032 | -0.043 | -0.021 | -3.48% | -4.67% | -2.28% | 0.000 |
| MCI/AD | K1-50% | -0.062 | -0.206 | 0.082 | -3.30% | -10.97% | 4.37% | 0.399 |
| MCI/AD | K1-25% | -0.010 | -0.062 | 0.042 | -0.52% | -3.28% | 2.23% | 0.710 |
| MCI/AD | K1-10% | -0.001 | -0.018 | 0.017 | -0.03% | -0.97% | 0.91% | 0.952 |
| MCI/AD | K1+10% | 0.002 | -0.012 | 0.017 | 0.13% | -0.64% | 0.90% | 0.739 |
| MCI/AD | K1+25% | 0.001 | -0.030 | 0.033 | 0.08% | -1.62% | 1.77% | 0.930 |
| MCI/AD | K1+50% | -0.003 | -0.056 | 0.050 | -0.15% | -2.97% | 2.67% | 0.916 |

Supplementary Table 16: Bias on SUVR_90_ due to perfusion changes with variable R1

| Diagnosis | K1 change | Region | Difference | 95% CI | | Relative difference | Relative 95% CI | | P value |
| --- | --- | --- | --- | --- | --- | --- | --- | --- | --- |
|  |  |  |  | lower | upper |  | lower | upper |  |
| HC | K1-50% | VOI 1 | 0.085 | 0.045 | 0.125 | 9.36% | 4.95% | 13.78% | 0.000 |
| HC | K1-25% | VOI 1 | 0.031 | 0.017 | 0.045 | 3.42% | 1.89% | 4.96% | 0.000 |
| HC | K1-10% | VOI 1 | 0.011 | 0.006 | 0.015 | 1.17% | 0.66% | 1.68% | 0.000 |
| HC | K1+10% | VOI 1 | -0.009 | -0.013 | -0.005 | -0.97% | -1.39% | -0.54% | 0.000 |
| HC | K1+25% | VOI 1 | -0.019 | -0.028 | -0.011 | -2.15% | -3.07% | -1.22% | 0.000 |
| HC | K1+50% | VOI 1 | -0.033 | -0.046 | -0.019 | -3.61% | -5.14% | -2.08% | 0.000 |
| HC | K1-50% | VOI 2 | 0.123 | 0.079 | 0.167 | 14.89% | 9.59% | 20.19% | 0.000 |
| HC | K1-25% | VOI 2 | 0.042 | 0.027 | 0.057 | 5.09% | 3.28% | 6.90% | 0.000 |
| HC | K1-10% | VOI 2 | 0.014 | 0.009 | 0.019 | 1.68% | 1.08% | 2.28% | 0.000 |
| HC | K1+10% | VOI 2 | -0.012 | -0.015 | -0.008 | -1.39% | -1.86% | -0.93% | 0.000 |
| HC | K1+25% | VOI 2 | -0.025 | -0.033 | -0.017 | -3.03% | -4.06% | -2.01% | 0.000 |
| HC | K1+50% | VOI 2 | -0.041 | -0.055 | -0.027 | -5.00% | -6.68% | -3.32% | 0.000 |
| HC | K1-50% | VOI 3 | 0.090 | 0.052 | 0.129 | 9.75% | 5.58% | 13.91% | 0.000 |
| HC | K1-25% | VOI 3 | 0.032 | 0.019 | 0.046 | 3.48% | 2.04% | 4.92% | 0.000 |
| HC | K1-10% | VOI 3 | 0.011 | 0.006 | 0.015 | 1.18% | 0.70% | 1.66% | 0.000 |
| HC | K1+10% | VOI 3 | -0.009 | -0.013 | -0.005 | -0.97% | -1.36% | -0.58% | 0.000 |
| HC | K1+25% | VOI 3 | -0.020 | -0.028 | -0.012 | -2.15% | -3.01% | -1.30% | 0.000 |
| HC | K1+50% | VOI 3 | -0.033 | -0.046 | -0.020 | -3.61% | -5.01% | -2.20% | 0.000 |
| HC | K1-50% | VOI 3+ | 0.069 | 0.041 | 0.097 | 6.81% | 4.04% | 9.58% | 0.000 |
| HC | K1-25% | VOI 3+ | 0.027 | 0.017 | 0.036 | 2.63% | 1.65% | 3.60% | 0.000 |
| HC | K1-10% | VOI 3+ | 0.009 | 0.006 | 0.013 | 0.92% | 0.59% | 1.24% | 0.000 |
| HC | K1+10% | VOI 3+ | -0.008 | -0.011 | -0.005 | -0.76% | -1.04% | -0.48% | 0.000 |
| HC | K1+25% | VOI 3+ | -0.017 | -0.024 | -0.011 | -1.72% | -2.33% | -1.11% | 0.000 |
| HC | K1+50% | VOI 3+ | -0.030 | -0.040 | -0.019 | -2.92% | -3.94% | -1.91% | 0.000 |
| HC | K1-50% | VOI 4 | 0.080 | 0.045 | 0.114 | 8.05% | 4.58% | 11.52% | 0.000 |
| HC | K1-25% | VOI 4 | 0.028 | 0.017 | 0.040 | 2.87% | 1.69% | 4.06% | 0.000 |
| HC | K1-10% | VOI 4 | 0.010 | 0.006 | 0.014 | 0.98% | 0.58% | 1.37% | 0.000 |
| HC | K1+10% | VOI 4 | -0.008 | -0.011 | -0.005 | -0.81% | -1.13% | -0.48% | 0.000 |
| HC | K1+25% | VOI 4 | -0.018 | -0.025 | -0.011 | -1.79% | -2.50% | -1.08% | 0.000 |
| HC | K1+50% | VOI 4 | -0.030 | -0.041 | -0.018 | -3.00% | -4.18% | -1.83% | 0.000 |
| HC | K1-50% | VOI 5 | 0.083 | 0.057 | 0.108 | 8.84% | 6.10% | 11.57% | 0.000 |
| HC | K1-25% | VOI 5 | 0.029 | 0.020 | 0.037 | 3.09% | 2.18% | 4.00% | 0.000 |
| HC | K1-10% | VOI 5 | 0.010 | 0.007 | 0.013 | 1.04% | 0.74% | 1.34% | 0.000 |
| HC | K1+10% | VOI 5 | -0.008 | -0.010 | -0.006 | -0.86% | -1.10% | -0.62% | 0.000 |
| HC | K1+25% | VOI 5 | -0.018 | -0.023 | -0.013 | -1.90% | -2.43% | -1.37% | 0.000 |
| HC | K1+50% | VOI 5 | -0.030 | -0.038 | -0.022 | -3.18% | -4.05% | -2.30% | 0.000 |
| HC | K1-50% | VOI 6 | 0.080 | 0.057 | 0.103 | 9.12% | 6.45% | 11.78% | 0.000 |
| HC | K1-25% | VOI 6 | 0.028 | 0.020 | 0.036 | 3.19% | 2.29% | 4.09% | 0.000 |
| HC | K1-10% | VOI 6 | 0.009 | 0.007 | 0.012 | 1.08% | 0.77% | 1.38% | 0.000 |
| HC | K1+10% | VOI 6 | -0.008 | -0.010 | -0.006 | -0.89% | -1.13% | -0.65% | 0.000 |
| HC | K1+25% | VOI 6 | -0.017 | -0.022 | -0.013 | -1.96% | -2.50% | -1.43% | 0.000 |
| HC | K1+50% | VOI 6 | -0.029 | -0.037 | -0.021 | -3.28% | -4.17% | -2.39% | 0.000 |
| MCI/AD | K1-50% | VOI 1 | -0.093 | -0.243 | 0.058 | -4.47% | -11.73% | 2.79% | 0.227 |
| MCI/AD | K1-25% | VOI 1 | -0.017 | -0.075 | 0.041 | -0.80% | -3.59% | 1.98% | 0.572 |
| MCI/AD | K1-10% | VOI 1 | -0.002 | -0.022 | 0.018 | -0.09% | -1.06% | 0.89% | 0.860 |
| MCI/AD | K1+10% | VOI 1 | 0.005 | -0.012 | 0.022 | 0.23% | -0.60% | 1.06% | 0.585 |
| MCI/AD | K1+25% | VOI 1 | 0.005 | -0.033 | 0.043 | 0.24% | -1.59% | 2.08% | 0.794 |
| MCI/AD | K1+50% | VOI 1 | 0.002 | -0.062 | 0.066 | 0.08% | -3.01% | 3.18% | 0.957 |
| MCI/AD | K1-50% | VOI 2 | 0.041 | -0.101 | 0.183 | 2.55% | -6.22% | 11.31% | 0.569 |
| MCI/AD | K1-25% | VOI 2 | 0.029 | -0.024 | 0.082 | 1.80% | -1.47% | 5.07% | 0.281 |
| MCI/AD | K1-10% | VOI 2 | 0.013 | -0.005 | 0.031 | 0.79% | -0.33% | 1.91% | 0.169 |
| MCI/AD | K1+10% | VOI 2 | -0.009 | -0.024 | 0.007 | -0.52% | -1.48% | 0.43% | 0.283 |
| MCI/AD | K1+25% | VOI 2 | -0.023 | -0.057 | 0.012 | -1.39% | -3.49% | 0.71% | 0.194 |
| MCI/AD | K1+50% | VOI 2 | -0.042 | -0.099 | 0.015 | -2.60% | -6.12% | 0.91% | 0.147 |
| MCI/AD | K1-50% | VOI 3 | -0.167 | -0.373 | 0.040 | -7.21% | -16.18% | 1.75% | 0.115 |
| MCI/AD | K1-25% | VOI 3 | -0.043 | -0.122 | 0.037 | -1.84% | -5.28% | 1.59% | 0.293 |
| MCI/AD | K1-10% | VOI 3 | -0.010 | -0.038 | 0.017 | -0.44% | -1.65% | 0.76% | 0.468 |
| MCI/AD | K1+10% | VOI 3 | 0.012 | -0.012 | 0.035 | 0.51% | -0.50% | 1.52% | 0.322 |
| MCI/AD | K1+25% | VOI 3 | 0.020 | -0.032 | 0.072 | 0.86% | -1.39% | 3.11% | 0.453 |
| MCI/AD | K1+50% | VOI 3 | 0.025 | -0.063 | 0.113 | 1.09% | -2.71% | 4.90% | 0.573 |
| MCI/AD | K1-50% | VOI 3+ | -0.104 | -0.240 | 0.031 | -5.15% | -11.83% | 1.54% | 0.131 |
| MCI/AD | K1-25% | VOI 3+ | -0.018 | -0.066 | 0.030 | -0.89% | -3.25% | 1.47% | 0.461 |
| MCI/AD | K1-10% | VOI 3+ | -0.002 | -0.018 | 0.015 | -0.08% | -0.88% | 0.72% | 0.837 |
| MCI/AD | K1+10% | VOI 3+ | 0.004 | -0.010 | 0.018 | 0.20% | -0.50% | 0.90% | 0.581 |
| MCI/AD | K1+25% | VOI 3+ | 0.003 | -0.028 | 0.033 | 0.12% | -1.39% | 1.64% | 0.872 |
| MCI/AD | K1+50% | VOI 3+ | -0.005 | -0.056 | 0.047 | -0.23% | -2.76% | 2.31% | 0.862 |
| MCI/AD | K1-50% | VOI 4 | -0.184 | -0.503 | 0.136 | -7.99% | -21.90% | 5.92% | 0.260 |
| MCI/AD | K1-25% | VOI 4 | -0.057 | -0.172 | 0.057 | -2.49% | -7.48% | 2.49% | 0.327 |
| MCI/AD | K1-10% | VOI 4 | -0.017 | -0.056 | 0.022 | -0.74% | -2.43% | 0.95% | 0.389 |
| MCI/AD | K1+10% | VOI 4 | 0.017 | -0.015 | 0.048 | 0.72% | -0.63% | 2.08% | 0.296 |
| MCI/AD | K1+25% | VOI 4 | 0.033 | -0.035 | 0.101 | 1.43% | -1.54% | 4.40% | 0.346 |
| MCI/AD | K1+50% | VOI 4 | 0.050 | -0.062 | 0.162 | 2.17% | -2.71% | 7.04% | 0.384 |
| MCI/AD | K1-50% | VOI 5 | 0.021 | -0.091 | 0.133 | 1.34% | -5.86% | 8.54% | 0.716 |
| MCI/AD | K1-25% | VOI 5 | 0.015 | -0.019 | 0.049 | 0.93% | -1.25% | 3.11% | 0.402 |
| MCI/AD | K1-10% | VOI 5 | 0.006 | -0.004 | 0.017 | 0.40% | -0.27% | 1.07% | 0.244 |
| MCI/AD | K1+10% | VOI 5 | -0.005 | -0.013 | 0.004 | -0.30% | -0.86% | 0.26% | 0.291 |
| MCI/AD | K1+25% | VOI 5 | -0.012 | -0.030 | 0.006 | -0.78% | -1.92% | 0.37% | 0.185 |
| MCI/AD | K1+50% | VOI 5 | -0.023 | -0.050 | 0.005 | -1.46% | -3.24% | 0.33% | 0.110 |
| MCI/AD | K1-50% | VOI 6 | 0.051 | -0.021 | 0.123 | 4.03% | -1.69% | 9.75% | 0.167 |
| MCI/AD | K1-25% | VOI 6 | 0.022 | 0.002 | 0.042 | 1.74% | 0.14% | 3.35% | 0.033 |
| MCI/AD | K1-10% | VOI 6 | 0.008 | 0.002 | 0.014 | 0.64% | 0.15% | 1.13% | 0.011 |
| MCI/AD | K1+10% | VOI 6 | -0.007 | -0.011 | -0.002 | -0.53% | -0.88% | -0.19% | 0.002 |
| MCI/AD | K1+25% | VOI 6 | -0.016 | -0.025 | -0.007 | -1.24% | -1.95% | -0.53% | 0.001 |
| MCI/AD | K1+50% | VOI 6 | -0.027 | -0.041 | -0.014 | -2.17% | -3.23% | -1.10% | 0.000 |

Supplementary Table 17: Bias on SUVR_90_ from scanning protocol non-compliance; pooling target regions

| Diagnosis | Delay | Difference | 95% CI | | Relative difference | Relative 95% CI | | P value |
| --- | --- | --- | --- | --- | --- | --- | --- | --- |
|  |  |  | lower | upper |  | lower | upper |  |
| HC | 5min | -0.004 | -0.005 | -0.002 | -0.39% | -0.55% | -0.23% | 0.000 |
| HC | 10min | -0.007 | -0.010 | -0.004 | -0.77% | -1.09% | -0.45% | 0.000 |
| HC | 20min | -0.014 | -0.019 | -0.008 | -1.46% | -2.10% | -0.83% | 0.000 |
| MCI/AD | 5min | 0.030 | 0.007 | 0.054 | 1.62% | 0.38% | 2.86% | 0.010 |
| MCI/AD | 10min | 0.059 | 0.013 | 0.106 | 3.16% | 0.70% | 5.62% | 0.012 |
| MCI/AD | 20min | 0.113 | 0.022 | 0.204 | 6.01% | 1.16% | 10.86% | 0.015 |

Supplementary Table 18: Bias on SUVR_90_ from scanning protocol non-compliance

| Diagnosis | Delay | Region | Difference | 95% CI | | Relative difference | Relative 95% CI | | P value |
| --- | --- | --- | --- | --- | --- | --- | --- | --- | --- |
|  |  |  |  | lower | upper |  | lower | upper |  |
| HC | 5min | VOI 1 | -0.004 | -0.005 | -0.003 | -0.45% | -0.54% | -0.36% | 0.000 |
| HC | 10min | VOI 1 | -0.008 | -0.010 | -0.006 | -0.89% | -1.06% | -0.71% | 0.000 |
| HC | 20min | VOI 1 | -0.015 | -0.018 | -0.012 | -1.70% | -2.03% | -1.38% | 0.000 |
| HC | 5min | VOI 2 | -0.008 | -0.010 | -0.007 | -1.01% | -1.17% | -0.84% | 0.000 |
| HC | 10min | VOI 2 | -0.016 | -0.018 | -0.013 | -1.92% | -2.22% | -1.61% | 0.000 |
| HC | 20min | VOI 2 | -0.029 | -0.034 | -0.024 | -3.50% | -4.07% | -2.93% | 0.000 |
| HC | 5min | VOI 3 | -0.003 | -0.005 | -0.001 | -0.30% | -0.51% | -0.10% | 0.004 |
| HC | 10min | VOI 3 | -0.006 | -0.009 | -0.002 | -0.60% | -0.99% | -0.20% | 0.003 |
| HC | 20min | VOI 3 | -0.011 | -0.017 | -0.004 | -1.15% | -1.88% | -0.43% | 0.002 |
| HC | 5min | VOI 3+ | 0.002 | -0.004 | 0.007 | 0.15% | -0.42% | 0.71% | 0.606 |
| HC | 10min | VOI 3+ | 0.003 | -0.009 | 0.014 | 0.28% | -0.84% | 1.40% | 0.627 |
| HC | 20min | VOI 3+ | 0.005 | -0.017 | 0.027 | 0.50% | -1.69% | 2.69% | 0.654 |
| HC | 5min | VOI 4 | -0.002 | -0.002 | -0.001 | -0.16% | -0.21% | -0.10% | 0.000 |
| HC | 10min | VOI 4 | -0.003 | -0.004 | -0.002 | -0.31% | -0.43% | -0.20% | 0.000 |
| HC | 20min | VOI 4 | -0.006 | -0.009 | -0.004 | -0.63% | -0.86% | -0.39% | 0.000 |
| HC | 5min | VOI 5 | -0.005 | -0.007 | -0.003 | -0.52% | -0.71% | -0.32% | 0.000 |
| HC | 10min | VOI 5 | -0.009 | -0.013 | -0.006 | -1.01% | -1.40% | -0.62% | 0.000 |
| HC | 20min | VOI 5 | -0.018 | -0.025 | -0.011 | -1.93% | -2.69% | -1.17% | 0.000 |
| HC | 5min | VOI 6 | -0.006 | -0.007 | -0.004 | -0.63% | -0.85% | -0.40% | 0.000 |
| HC | 10min | VOI 6 | -0.011 | -0.015 | -0.007 | -1.22% | -1.66% | -0.78% | 0.000 |
| HC | 20min | VOI 6 | -0.020 | -0.028 | -0.013 | -2.33% | -3.18% | -1.47% | 0.000 |
| MCI/AD | 5min | VOI 1 | 0.036 | 0.014 | 0.058 | 1.74% | 0.69% | 2.80% | 0.001 |
| MCI/AD | 10min | VOI 1 | 0.071 | 0.026 | 0.115 | 3.40% | 1.27% | 5.54% | 0.002 |
| MCI/AD | 20min | VOI 1 | 0.134 | 0.044 | 0.224 | 6.46% | 2.11% | 10.82% | 0.004 |
| MCI/AD | 5min | VOI 2 | 0.016 | -0.008 | 0.041 | 1.02% | -0.46% | 2.50% | 0.178 |
| MCI/AD | 10min | VOI 2 | 0.032 | -0.016 | 0.080 | 1.98% | -0.99% | 4.95% | 0.191 |
| MCI/AD | 20min | VOI 2 | 0.061 | -0.035 | 0.157 | 3.74% | -2.19% | 9.68% | 0.216 |
| MCI/AD | 5min | VOI 3 | 0.046 | 0.017 | 0.074 | 1.99% | 0.76% | 3.22% | 0.002 |
| MCI/AD | 10min | VOI 3 | 0.090 | 0.033 | 0.147 | 3.89% | 1.41% | 6.37% | 0.002 |
| MCI/AD | 20min | VOI 3 | 0.172 | 0.056 | 0.287 | 7.43% | 2.42% | 12.43% | 0.004 |
| MCI/AD | 5min | VOI 3+ | 0.034 | 0.014 | 0.055 | 1.69% | 0.69% | 2.69% | 0.001 |
| MCI/AD | 10min | VOI 3+ | 0.066 | 0.026 | 0.107 | 3.28% | 1.28% | 5.28% | 0.001 |
| MCI/AD | 20min | VOI 3+ | 0.125 | 0.044 | 0.205 | 6.16% | 2.19% | 10.13% | 0.002 |
| MCI/AD | 5min | VOI 4 | 0.048 | -0.001 | 0.096 | 2.08% | -0.03% | 4.19% | 0.053 |
| MCI/AD | 10min | VOI 4 | 0.094 | -0.002 | 0.190 | 4.09% | -0.09% | 8.26% | 0.055 |
| MCI/AD | 20min | VOI 4 | 0.181 | -0.007 | 0.369 | 7.88% | -0.31% | 16.07% | 0.059 |
| MCI/AD | 5min | VOI 5 | 0.020 | -0.004 | 0.044 | 1.27% | -0.27% | 2.81% | 0.107 |
| MCI/AD | 10min | VOI 5 | 0.038 | -0.008 | 0.085 | 2.47% | -0.53% | 5.47% | 0.107 |
| MCI/AD | 20min | VOI 5 | 0.073 | -0.016 | 0.162 | 4.68% | -1.02% | 10.38% | 0.108 |
| MCI/AD | 5min | VOI 6 | 0.012 | -0.007 | 0.032 | 0.98% | -0.58% | 2.55% | 0.219 |
| MCI/AD | 10min | VOI 6 | 0.024 | -0.014 | 0.062 | 1.90% | -1.12% | 4.92% | 0.218 |
| MCI/AD | 20min | VOI 6 | 0.045 | -0.026 | 0.116 | 3.55% | -2.05% | 9.15% | 0.214 |
